# Supplementary material for: Acetylcholine deficiency disrupts extratelencephalic projection neurons in the prefrontal cortex in a mouse model of Alzheimer’s disease
Source: Nat Commun. 2022 Feb 22;13:998. doi: 10.1038/s41467-022-28493-4 (PMC8863829; doi:10.1038/s41467-022-28493-4)
Supplement: Supplementary file 1 — Supplementary Information [file 41467_2022_28493_MOESM1_ESM.pdf]

Supplementary materials

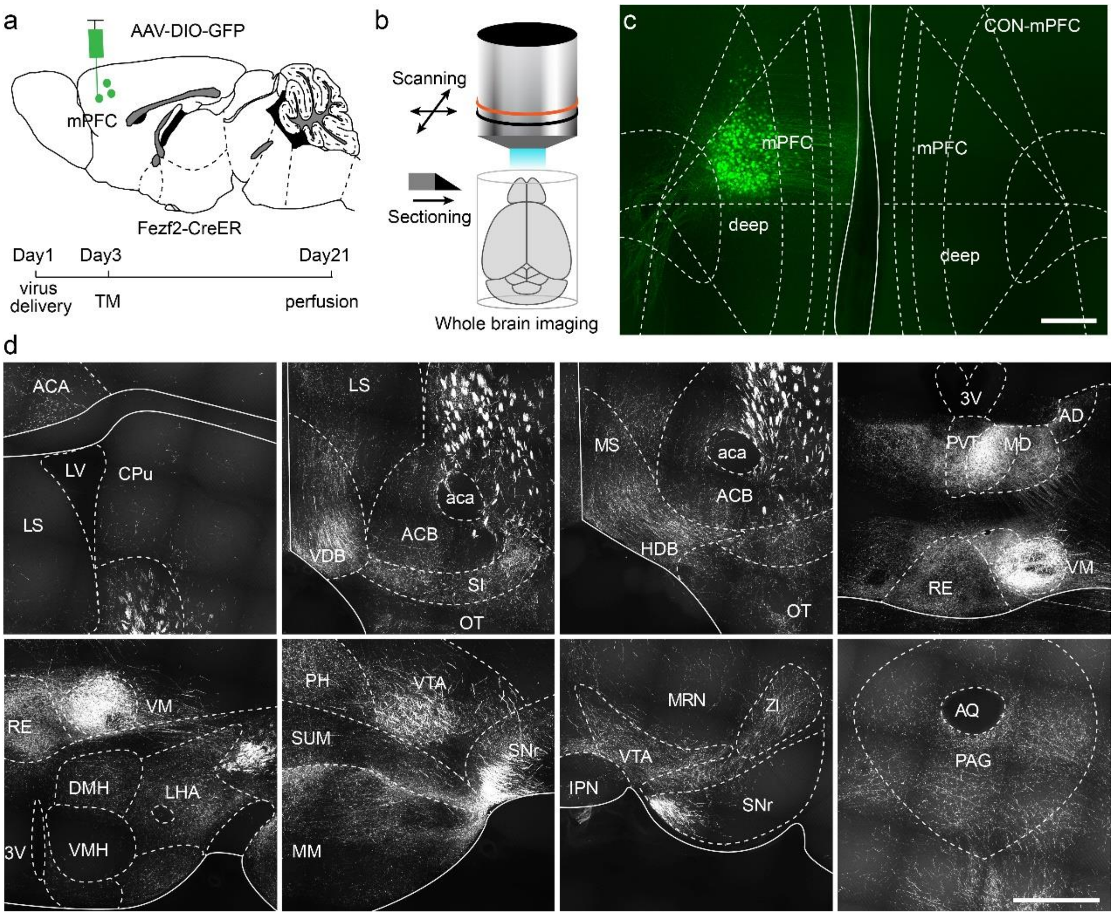

Supplementary figure 1 The output of extratelencephalic projection neurons in the mPFC, related to Fig 1. a. Labeling the Fezf2 positive extratelencephalic projection neurons in mPFC with AAV virus expressing GFP. b. The whole brain imaging process to acquire the output of Fezf2 positive neurons in mPFC. c. The virus injection site at mPFC. The infected neurons were mainly found in deep layers of mPFC, indicating the specificity of the virus labeling. d. The axon terminals of Fezf2 positive neurons in mPFC in multiple subcortical areas. Scale bar in c is 200 μm. Scale bar in d is 500 μm. TM, tamoxifen; mPFC, medial prefrontal cortex; ACA, anterior cingulate area; LV, lateral ventricle; LS, lateral septum; CPu, caudate putamen; ACB, nucleus accumbens; VDB, vertical diagonal band; SI, substantia innominata; OT, olfactory tubercle; MS, medial septum; aca, anterior commissure, anterior part; 3V, third ventricle; PVT, paraventricular thalamic nucleus; MD, mediodorsal thalamic nucleus; AD, anterodorsal thalamic nucleus; RE, reuniens thalamic nucleus; VM, ventromedial thalamic nucleus; DMH, dorsomedial hypothalamic nucleus; VMH, ventromedial hypothalamic nucleus; LHA, lateral hypothalamic area; PH, posterior hypothalamic area; SUM, supramammillary nucleus; VTA, ventral tegmental area; SNR, substantia nigra, reticular part; MM, medial mammillary nucleus; IPN, interpeduncular nucleus; ZI, zona incerta; MRN, midbrain reticular nucleus; PAG, periaqueductal gray; AQ, aqueduct.

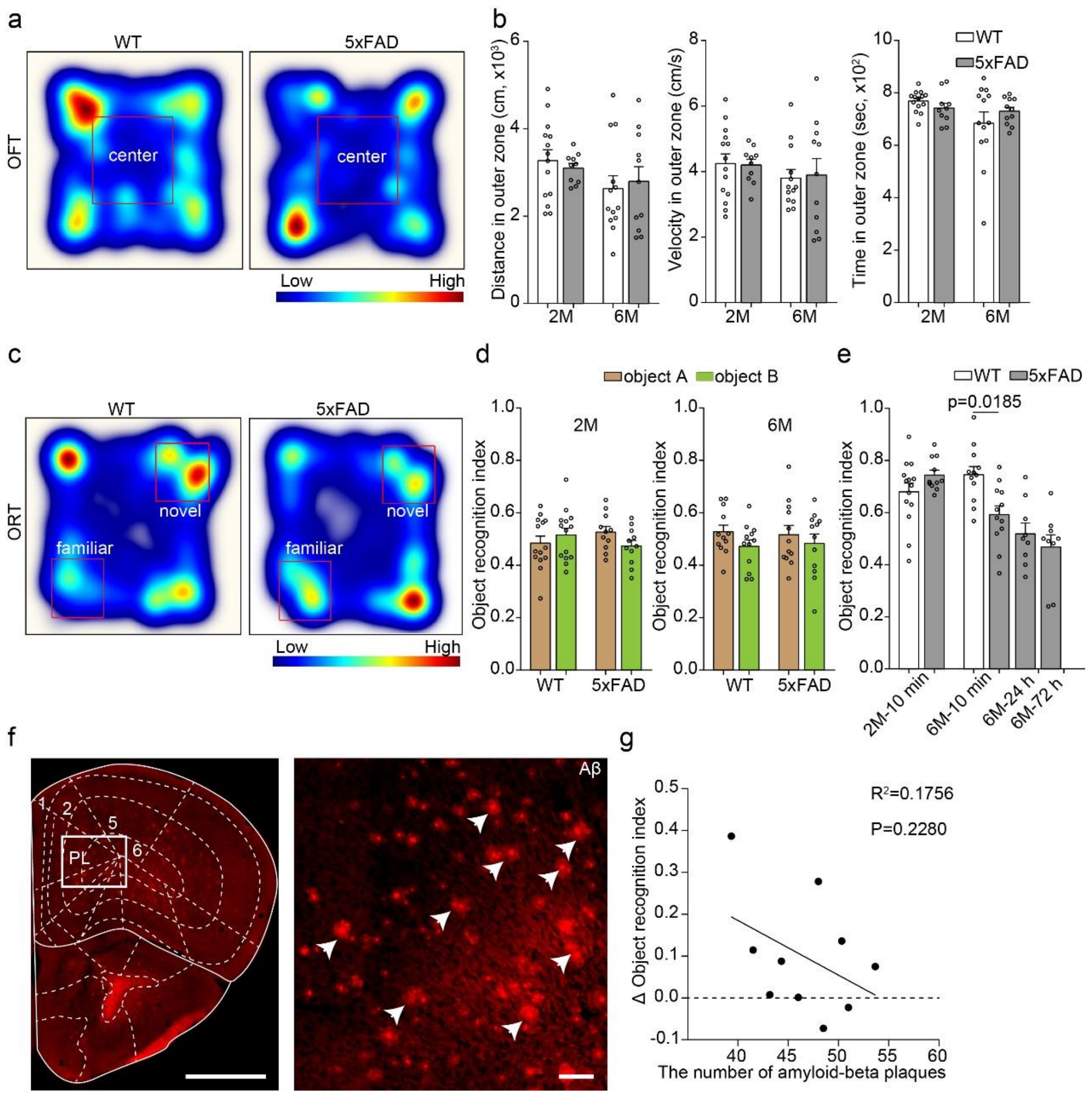

Supplementary figure 2. Recognition memory was impaired in AD mice, related to Fig 1. a. the locomotor activity was assessed in an open-field test. Heat-map plots showed the time that mice spent in outer zone during the test. Red = more time, blue = less time. b. there was no statistical difference in total distance traveled, time spent and velocity for 5×FAD compared to their wild-type littermates at 2 months and 6 months of age in outer of the arena. (2M, n = 14 animals for WT, n = 10 animals for AD; 6M, n = 13 animals for WT, n = 11 animals for AD). c. Heat-map plots showed object recognition memory measures from the object recognition test. Red = more time, blue = less time. d. There was no statistical difference in time spent to explore one of the objects during the training session for 5×FAD compared to their wild-type littermates at 2 months and 6 months of age, respectively. (2M, n = 14 animals for WT, n = 11 animals for AD; 6M, n = 12 animals for WT, n = 12 animals for AD) e. During the test session, the 5×FAD mice significantly spent less time exploring the novel object compared with the wild-type littermates at 6 months of age. With longer delay time, the 5×FAD animals showed worse performance during the object recognition test. (one-way repeated-measures (RM) ANOVA with Tukey's post hoc test, 2M, n = 14 animals for WT, n = 11 animals for AD; 6M, 10min delay, n = 12 animals for WT, n = 12 animals for AD; 6M, 24h and 72h delay, n=9 animals for AD). f.

Representative images showed the Aβ plagues accumulation in the mPFC. g. The correlation between delta recognition index and the density of Aβ plagues in the mPFC of 5×FAD mice at 6 months of age. n=10 animals. Linear regression analysis. Scale bar in the f(left) is 1mm; scale bar in the f(right) is 100 μm. All data are listed as the Mean ± SEM.

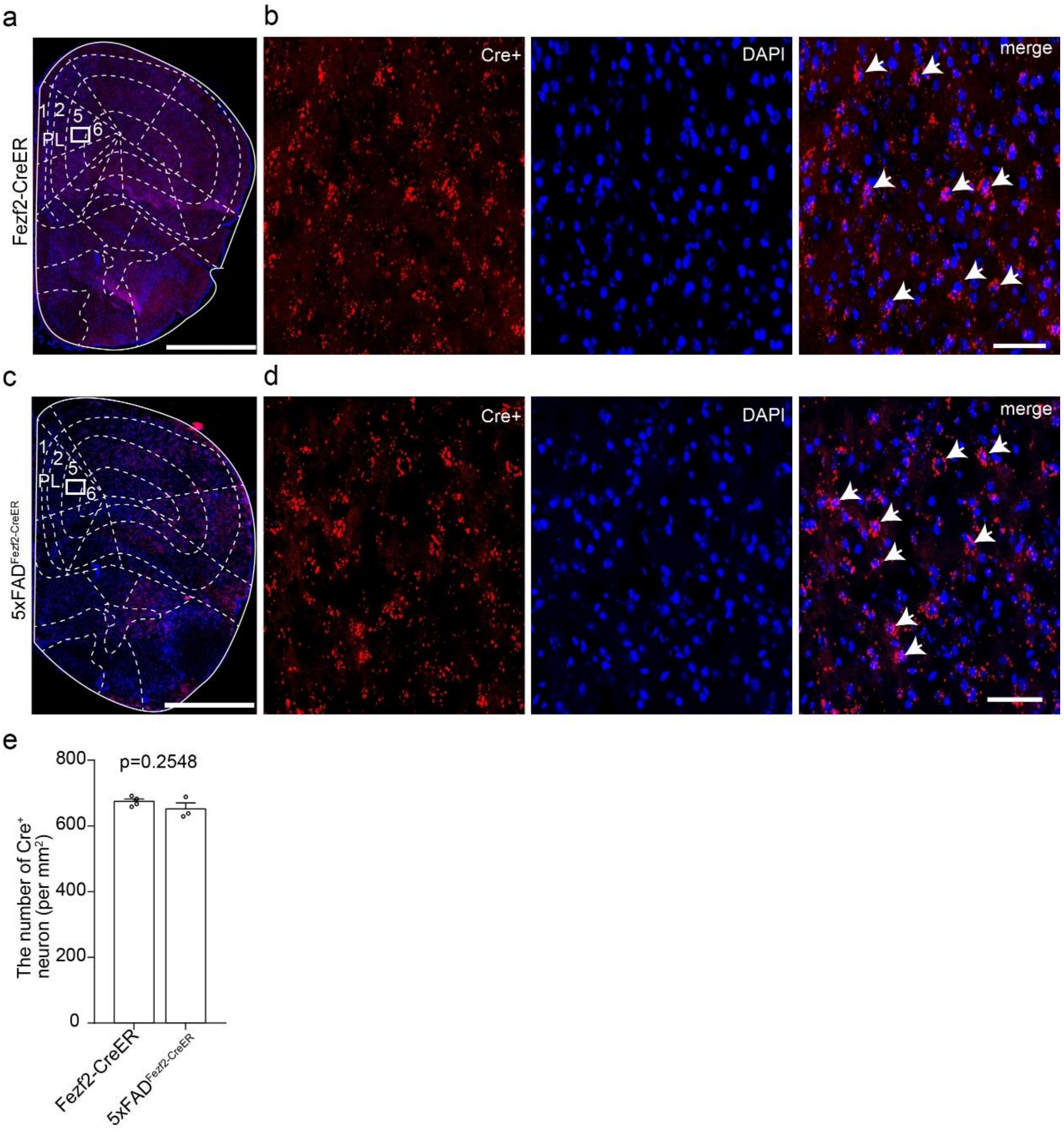

Supplementary figure 3 Crossing the 5×FAD animals with Fezf2-CreER animals did not alter the number of Cre positive cells in the mPFC. a-d. Fluorescent in situ hybridization against Cre showed the Cre expression in the mPFC of Fezf2-CreER animals and the 5×FAD$_{Fezf2\text{-}CreER}$ hybrid animals. e. Quantification of Cre positive cells in the mPFC of Fezf2-CreER animals and the 5×FAD $_{Fezf2\text{-}CreER}$ hybrid animals. Fezf2-CreER, n=4 animals; 5×FAD$_{Fezf2\text{-}CreER}$, n=3 animals, two-tailed unpaired t test. Scale bars in the a and c are 1mm; scale bars in the b and d are 100 μm. All data are listed as the Mean ± SEM.

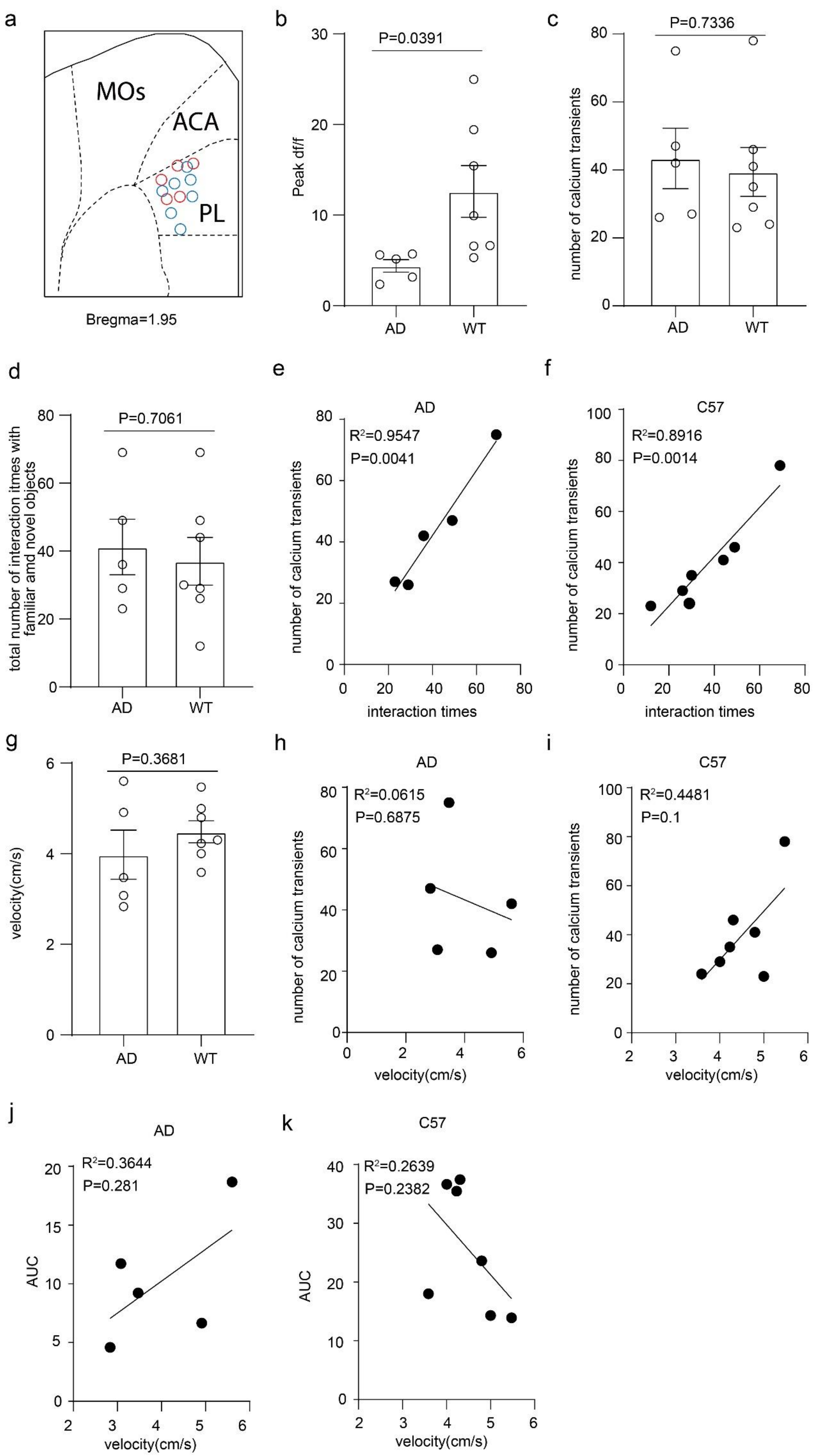
a
MOs
ACA
PL
Bregma=1.95
b
P=0.0391
Peak df/f
AD
WT
c
P=0.7336
number of calcium transients
AD
WT
d
P=0.7061
total number of interaction itmes with familiar amd novel objects
AD
WT
e
AD
R²=0.9547
P=0.0041
number of calcium transients
interaction times
f
C57
R²=0.8916
P=0.0014
number of calcium transients
interaction times
g
P=0.3681
velocity(cm/s)
AD
WT
h
AD
R²=0.0615
P=0.6875
number of calcium transients
velocity(cm/s)
i
C57
R²=0.4481
P=0.1
number of calcium transients
velocity(cm/s)
j
AD
R²=0.3644
P=0.281
AUC
velocity(cm/s)
k
C57
R²=0.2639
P=0.2382
AUC
velocity(cm/s)

Supplementary figure 4 The properties of the fiber photometry data from the ET neurons of Fezf2-CreER animals and the $AD_{Fezf2\text{-}CreER\ animals}$. a. Schematics showing the placement of optic fibers in Fig 1. The red circles represent the placement of optic fibers in the PL of $AD_{Fezf2\text{-}CreER}$ mice. The blue circles represent the placement of optic fibers in the PL of Fezf2-CreER mice. b. The quantification of peak delta/f of average plots of calcium response from different animals. c. The quantification of the number of calcium transients during the photometry recording of different animals. d. The total number of interaction times with familiar and novel objects of the animals during the object recognition test. e, f. The correlation between object interaction times and the number of calcium transients in Fezf2-CreER animals and the $AD_{Fezf2\text{-}CreER\ animals}$. g. The average velocity of the animals in object recognition test. h, i. The correlation between velocity and the number of calcium transients in Fezf2-CreER animals and the $AD_{Fezf2\text{-}CreER\ animals}$. j, k. The correlation between velocity and the AUC in Fezf2-CreER animals and the $AD_{Fezf2\text{-}CreER\ animals}$. Two-tailed unpaired t test, Fezf2-CreER, n=7 animals. $AD_{Fezf2\text{-}CreER\ animals,}$ n=5 animals. MOs, secondary motor area; ACA, anterior cingulate area; PL, prelimbic area. All data are listed as the Mean ± SEM.

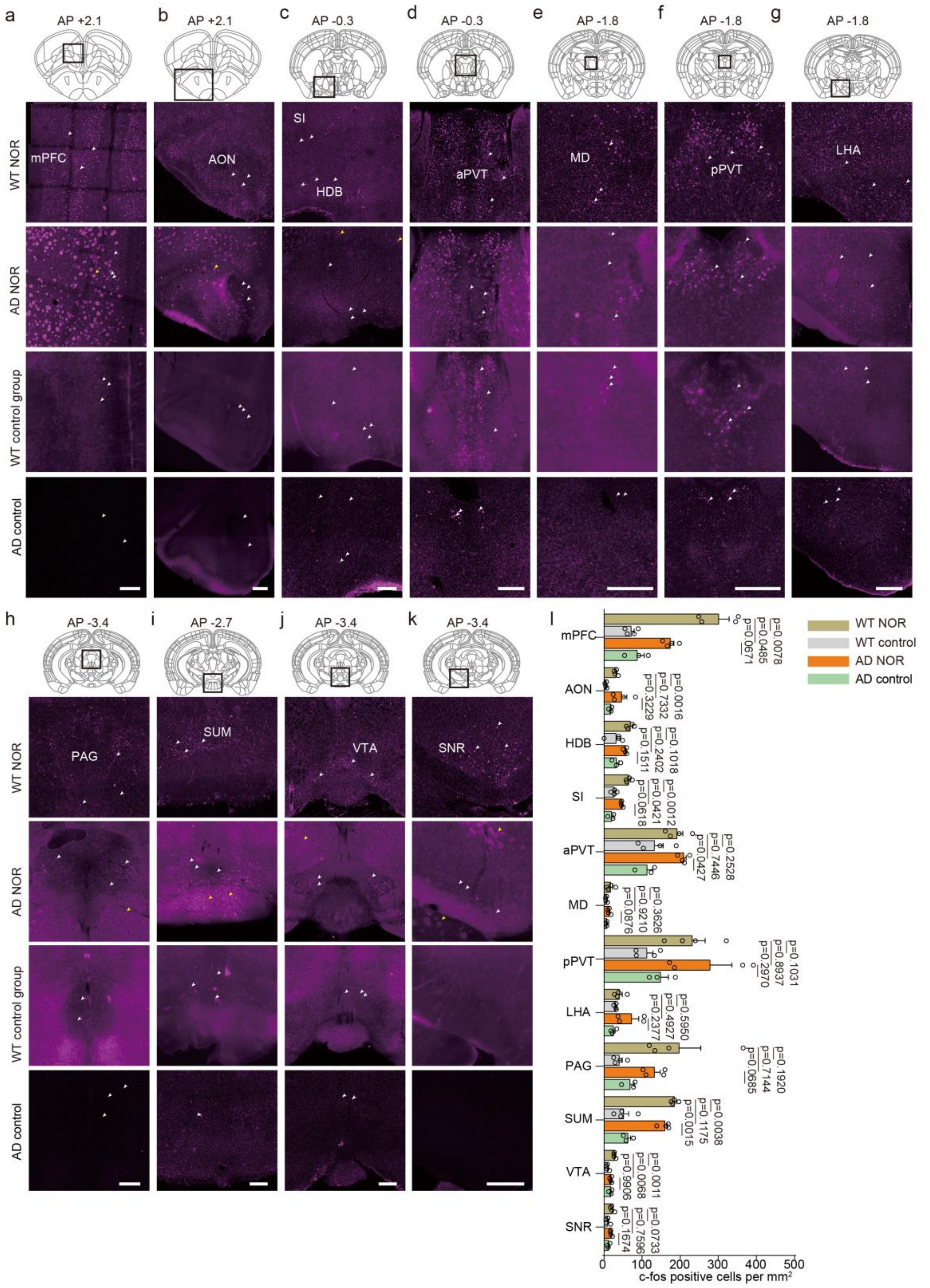

Supplementary figure 5 The altered neuronal activity activated by object exploration in downstream brain areas of extratelencephalic projection neurons in the mPFC of 5×FAD mice, related to Fig 1. a-k. Example c-fos immunostaining in downstream brain areas of extratelencephalic projection neurons in the mPFC of wild type mice or 5×FAD mice before and after object exploration. l. Counts of c-fos+ cells in different brain areas after object exploration. Two-way repeated-measures (RM) ANOVA with Tukey's post hoc test, n = 4

animals for WT object recognition test group, WT control group and AD object recognition test group. n=3 animals for AD control group. Scale bars in a-k are 100 μm. mPFC, medial prefrontal cortex; AON, anterior olfactory nucleus; HDB, horizontal diagonal band; SI, substantia innominata; aPVT, anterior paraventricular thalamic nucleus; MD, mediodorsal thalamic nucleus; pPVT, posterior paraventricular thalamic nucleus; LHA, lateral hypothalamic area; PAG, periaqueductal gray; SUM, supramammillary nucleus; VTA, ventral tegmental area; SNR, substantia nigra, reticular part. All data are listed as the Mean ± SEM.

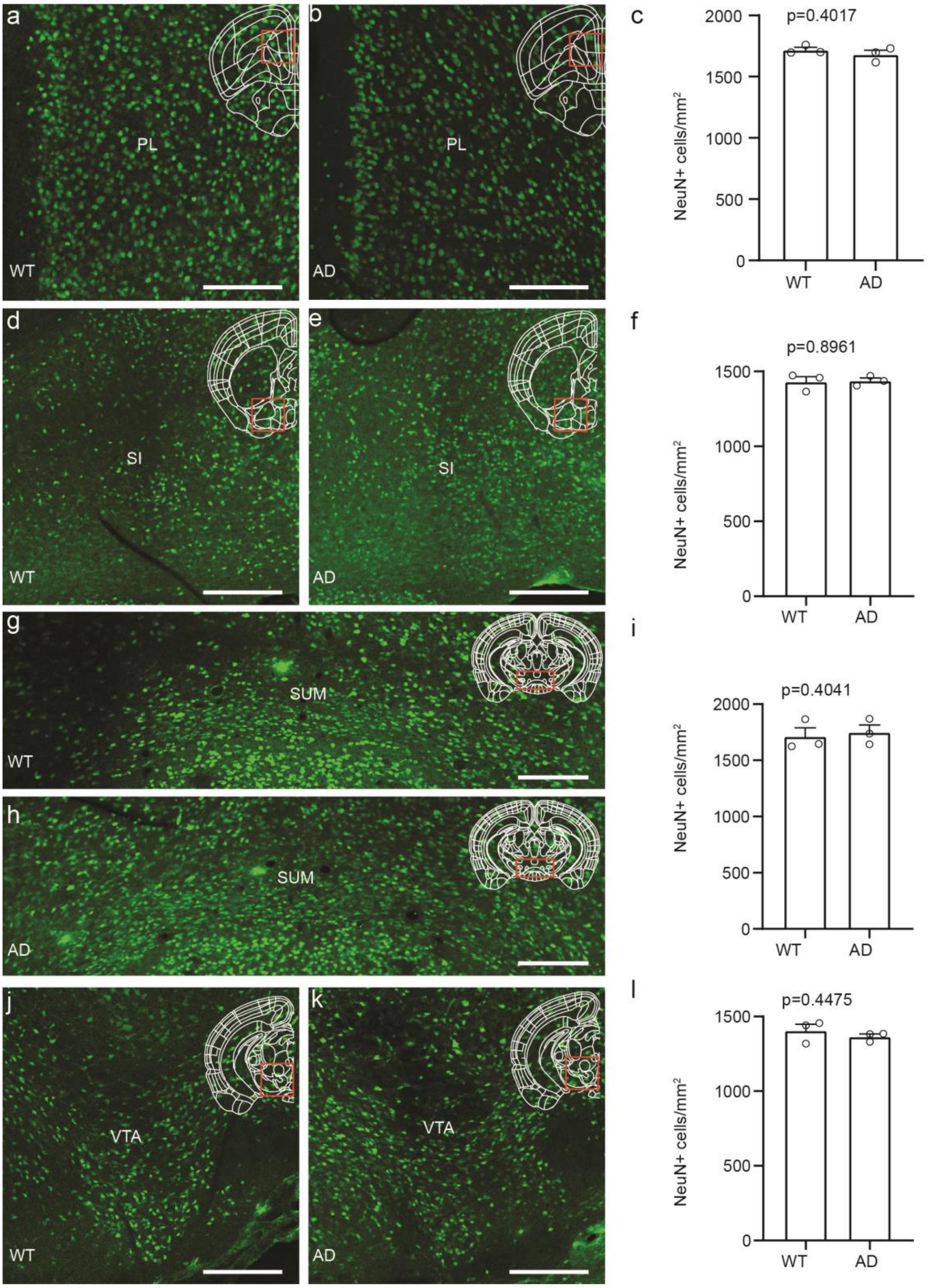

Supplementary figure 6 The NeuN density in the mPFC, SI, SUM AND VTA of WT animals and 5×FAD animals. a-c. The NeuN density showed no difference in the mPFC between WT animals and 5×FAD animals. d-f. The NeuN density showed no difference in the SI between WT animals and 5×FAD animals. g-i. The NeuN density showed no difference in the SUM between WT animals and 5×FAD animals. j-l. The NeuN density showed no difference in the VTA between WT animals and 5×FAD animals. Two-tailed unpaired t test, n=3 animals. Scale

bars are 200 μm. mPFC, medial prefrontal cortex; SI, substantia innominata; SUM, supramammillary nucleus; VTA, ventral tegmental area. All data are listed as the Mean ± SEM.

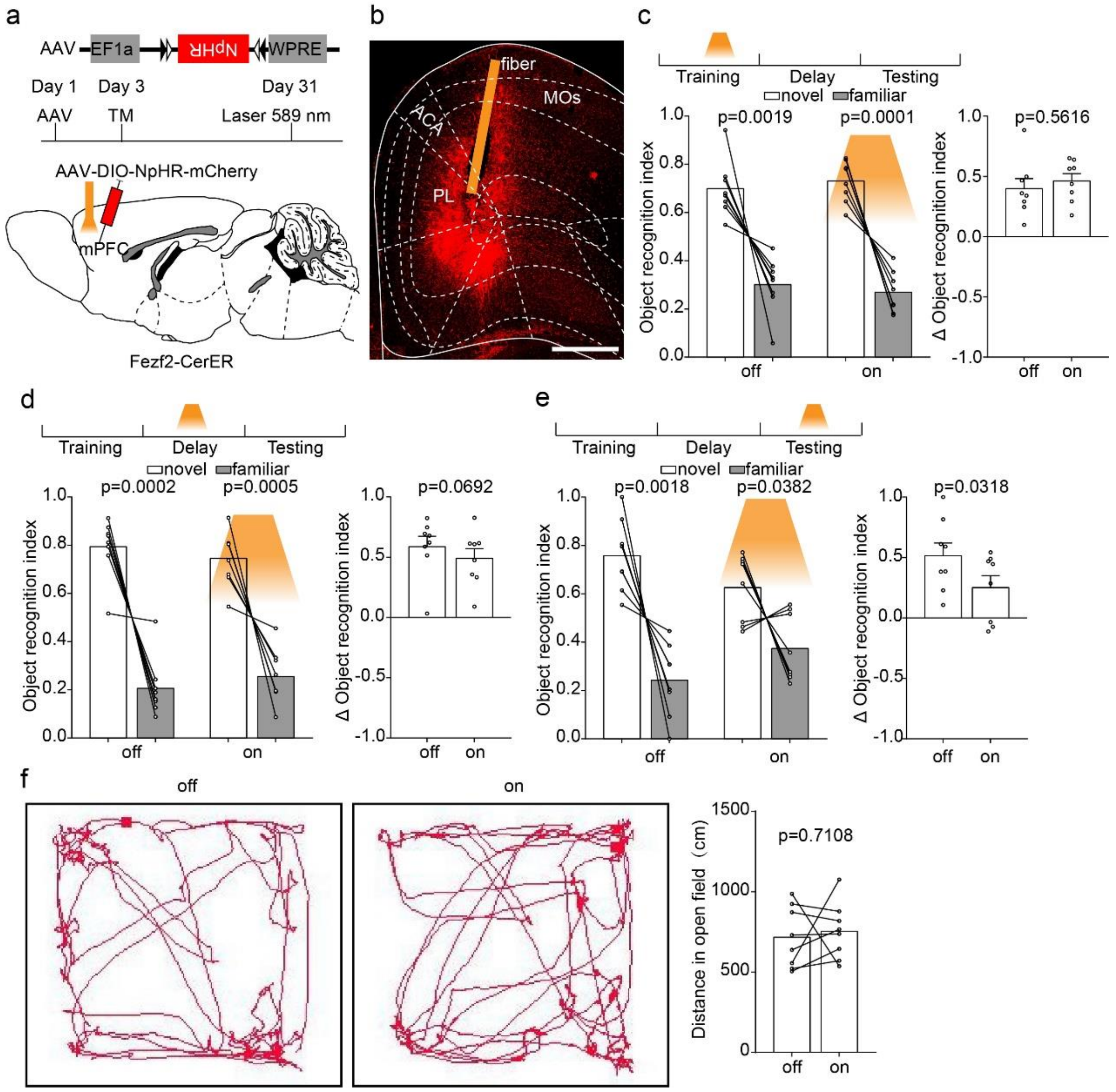

Supplementary figure 7 Inhibition of extratelencephalic projection neurons in the mPFC of wild type mice in specific phase disrupted object recognition memory expression, related to Fig 2. a. The experimental strategy of inhibition of extratelencephalic projection neurons in the mPFC. The Cre dependent AAV expressing NpHR was injected into the mPFC of Fezf2-CreER mice. Three days after the virus injection, tamoxifen (200mg/kg) was injected intraperitoneally to activate the expression of NpHR. 3-4 weeks after the virus injection, the behavior tests were performed. b. An example of position of optical fiber for inhibition of the extratelencephalic projection neurons in the mPFC. c. Statistical plots showed that the Fezf2-CreER mice tended to explore the novel object under two conditions (with or without light stimulation during training session, two-tailed paired t test). The delta object cognition index showed no difference under two conditions (two-tailed paired t test). n = 8 animals. d. Statistical plots showed that the Fezf2-CreER mice tended to explore the novel object under two conditions (with or without light stimulation during delay session, two-tailed paired t test). The delta object cognition index showed no difference under two conditions (two-tailed paired t test). n = 8 animals. e. Statistical plots showed that during the test session, the Fezf2-CreER mice that received light stimulation significantly spent less time exploring the novel object compared to the sessions without light

stimulation at 6 months of age, two-tailed paired t test. The animals which received light stimulation during test session showed decreased delta object recognition index, two-tailed paired t test, n = 8 animals. f. Inhibition of extratelencephalic projection neurons in the mPFC did not affect the locomotion of the animals. f(left), the representative traces of the animals during the open field test with or without light inhibition. f(right), the quantification of the traveling distance of the animals with or without light inhibition. two-tailed paired t test, n=8 animals. Scale bar in b is 500 μm. TM, tamoxifen; MOs, secondary motor area; ACA, anterior cingulate area; PL, prelimbic area. All data are listed as the Mean ± SEM.

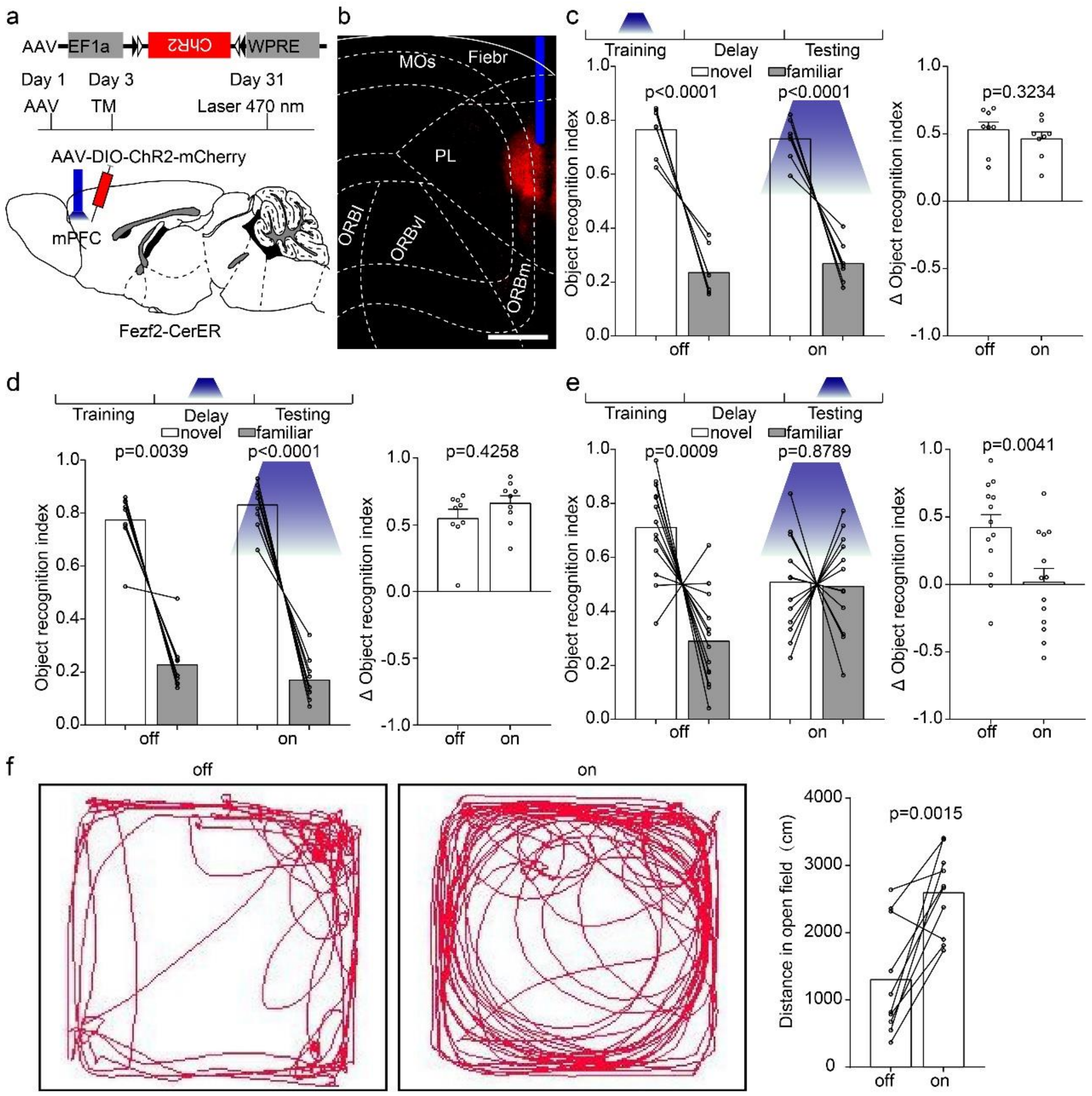

Supplementary figure 8 Activation of extratelencephalic projection neurons in the mPFC of Fezf2-CreER mice in specific phase disrupted object recognition memory expression, related to Fig 2. a. The experimental strategy of activation of extratelencephalic projection neurons in the mPFC. The Cre dependent AAV expressing ChR2 was injected into the mPFC of Fezf2-CreER mice. Three days after the virus injection, tamoxifen (200mg/kg) was injected intraperitoneally to activate the expression of ChR2. 3-4 weeks after the virus injection, the behavior tests were performed. b. An example of position of optical fiber for activation of the extratelencephalic projection neurons in the mPFC. c. Statistical plots showed that the Fezf2-CreER mice tended to explore the novel object under two conditions (with or without light stimulation during training session, two-tailed paired t test, off, p=0.000041; on, p=0.000041). The delta object cognition index showed no difference under two conditions. two-tailed paired t test, n = 8 animals. d. Statistical plots showed that the Fezf2-CreER mice tended to explore the novel object under two conditions (with or without light stimulation during delay session, left, two-tailed Wilcoxon matched-pairs signed rank test; right, two-tailed paired t test, $p=2.38\times10^{-6}$). The delta object cognition index showed no difference under two conditions. two-tailed Wilcoxon matched-pairs signed rank test, n = 9 animals. e. Statistical plots showed

that during the test session, the Fezf2-CreER mice that received light stimulation significantly spent less time exploring the novel object compared to the sessions without light stimulation at 6 months of age (two-tailed paired t test). The animals which received light stimulation during test session showed decreased delta object recognition index, two-tailed paired t test, n = 13 animals. f. Activation of extratelencephalic projection neurons in the mPFC increased the locomotion of the animals. f(left), the representative traces of the animals during the open field test with or without light activation. f(right), the quantification of the traveling distance of the animals with or without light activation. two-tailed paired t test, n=10 animals. Scale bar in b is 500 μm. MOs, secondary motor area; PL, prelimbic area; ORBm, medial orbital cortex; ORBvl, ventrolateral orbital cortex; ORBl, lateral orbital cortex. All data are listed as the Mean ± SEM.

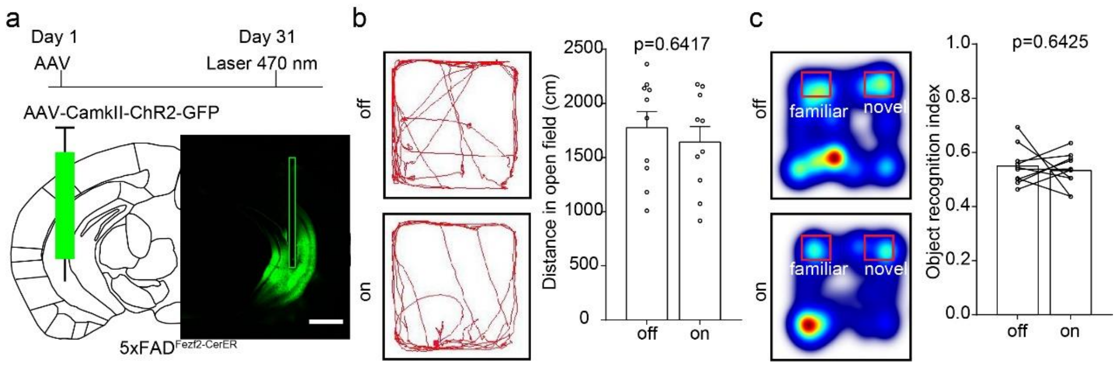

Supplementary figure 9 Activation of ventral hippocampus of 5×FAD mice did not improve the expression of object recognition memory. a. The experiment procedure. The AAV virus expressing ChR2 under the control of camkii promotor was bilaterally injected into the ventral hippocampus of 5×FAD mice. The behavior test was conducted 1 month after the surgery. b(left), the representative traces of the animals during the open field test with or without light activation. b(right), the quantification of the traveling distance of the animals with or without light activation. two-tailed paired t test, n=10 animals. c(left), Heat-map plots showed object recognition memory measures from the object recognition test. Red = more time, blue = less time. c(right), quantification of the object recognition index towards novel object with or without light activation. Activation of ventral hippocampus of 5×FAD mice did not increase the exploration time towards novel object. two-tailed paired t test, n=10 animals. Scale bar in a is 1mm. All data are listed as the Mean ± SEM.

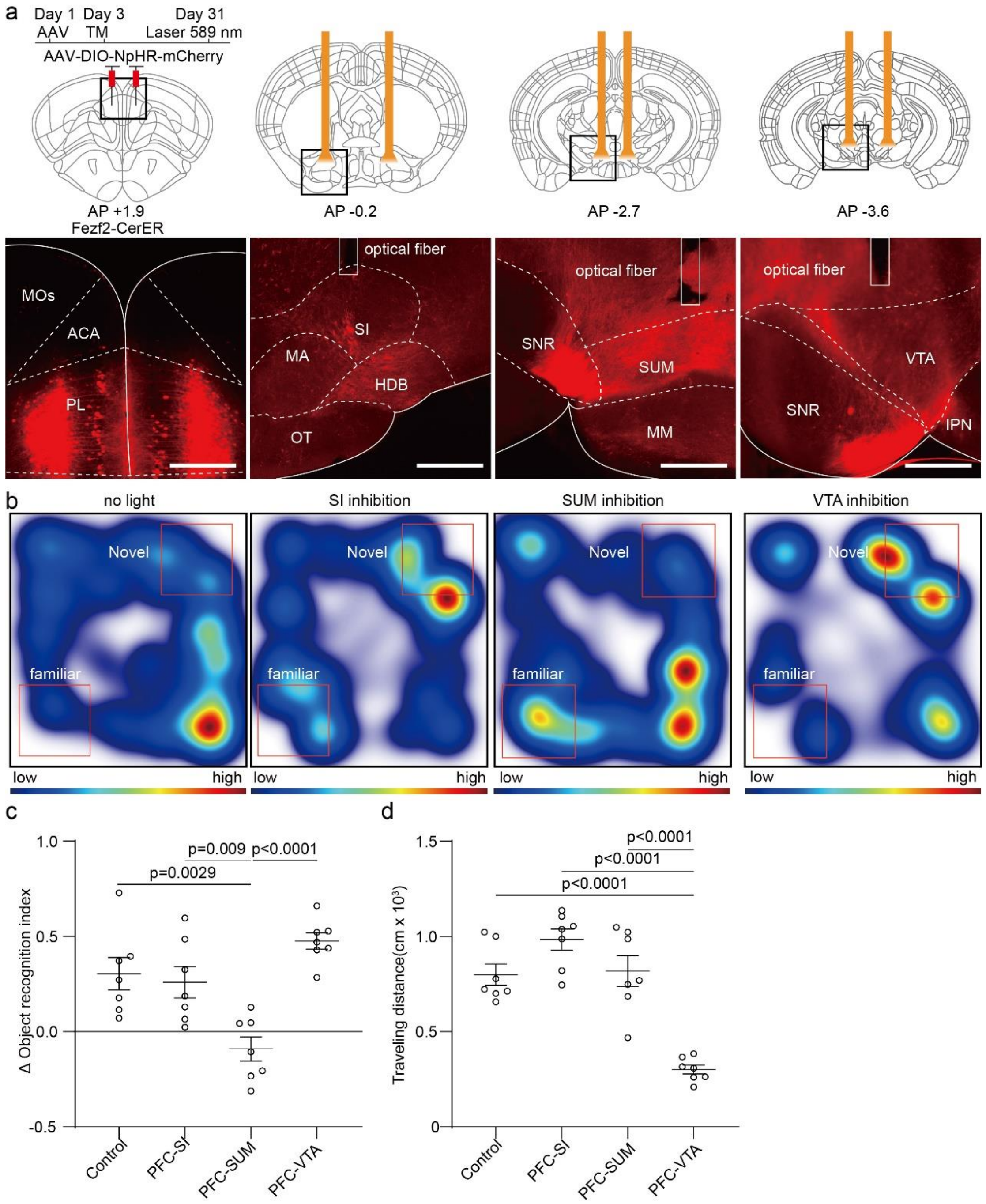

Supplementary figure 10 Inhibition of specific axon terminals of extratelencephalic projection neurons in the mPFC of Fezf2-CreER mice disrupted the object recognition memory, related to Fig 3. a. The experimental strategy of terminal inhibition of extratelencephalic projection neurons in the mPFC. The Cre dependent AAV expressing NpHR was injected into the mPFC of Fezf2-CreER mice. The optical fibers were implatnted in SI, SUM or VTA bilaterally to inhibit the axon terminals in these brain areas. Three days after the virus injection, tamoxifen (200 mg/kg) was injected intraperitoneally to activate the expression of NpHR. 3-4 weeks after the virus injection, the behavior tests were performed. The virus injection site and the positions of the optical fiber were shown below. b. Heat-map plots showed object recognition memory measures from the object recognition test under different experimental conditions (no light

stimulation; inhibition of axon terminals in SI; inhibition of axon terminals in SUM; inhibition of axon terminals in VTA.) Red = more time, blue = less time. c. Statistical plots showed the delta object recognition index under different conditions. One-way repeated-measures (RM) ANOVA with Tukey's post hoc test, PFC-SUM vs PFC-VTA, $p = 0.00004$. $n = 7$ animals. d. Statistical plots showed the traveling distance during the test session under different conditions. $n = 7$ animals. One-way repeated-measures (RM) ANOVA with Tukey's post hoc test, control vs PFC-VTA, $p = 0.000015$; PFC-SI vs PFC-VTA, $p = 0.00000008043$; PFC-SUM vs PFC-VTA, $p = 0.00000835702$. Scale bars in a is 500 μm. MOs, secondary motor area; ACA, anterior cingulate area; PL, prelimbic area; SI, substantia innominata; MA, magnocellular nucleus; HDB, horizontal diagonal band; OT, olfactory tubercle; SNR, substantia nigra, reticular part; SUM, supramammillary nucleus; MM, medial mammillary nucleus; VTA, ventral tegmental area; IPN, interpeduncular nucleus. All data are listed as the Mean ± SEM.

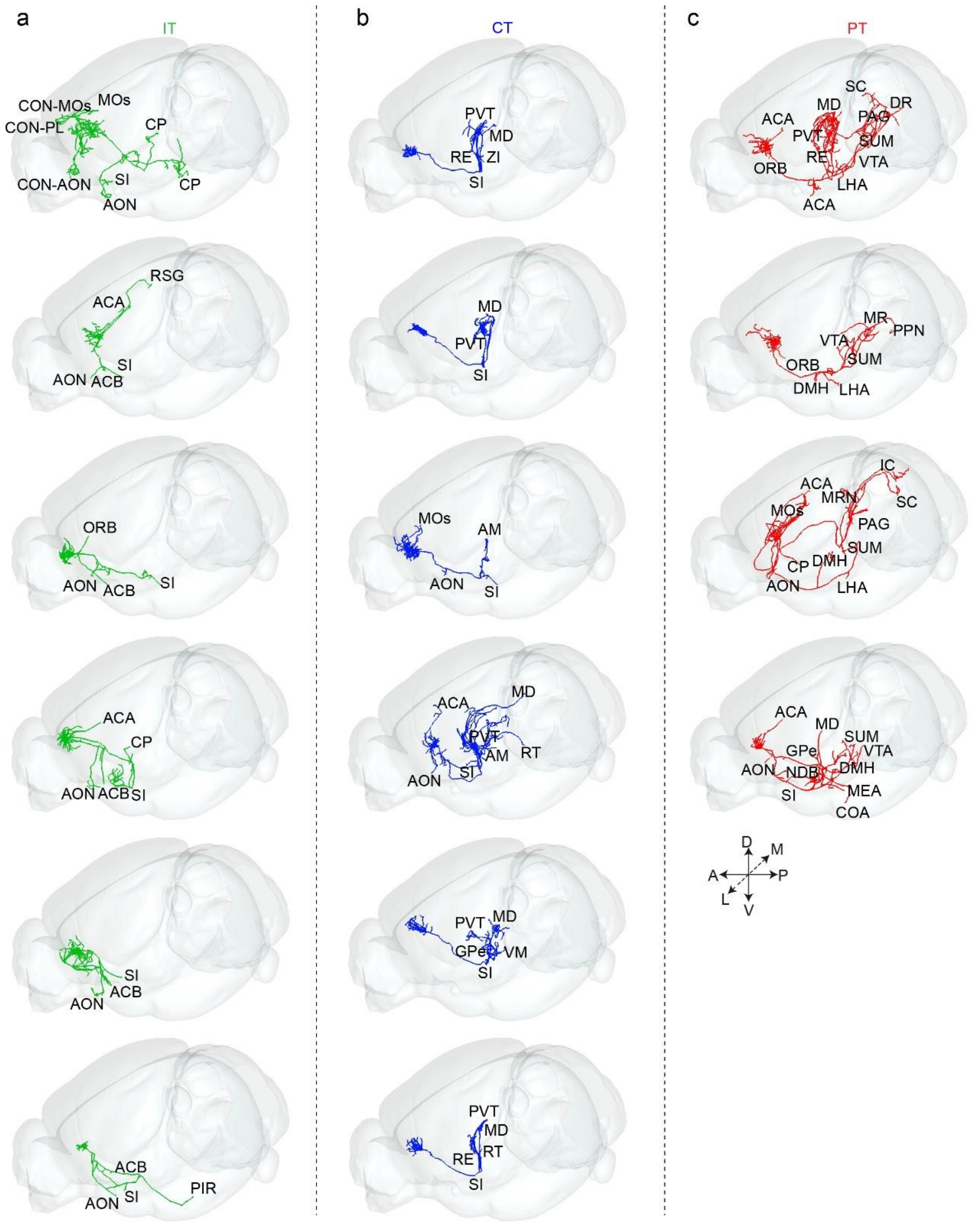

Supplementary figure 11 The morphology of three types of reconstructed extratelencephalic projection neurons in the mPFC, related to Fig 4. a-c. The CT neurons were shown in blue. The IT neurons were shown in green. The PT neurons were shown in red. MOs, secondary motor area; AON, anterior olfactory nucleus; CP, caudate putamen; SI, substantia innominata. ACB, nucleus accumbens; ACA, anterior cingulate area; RSG, retrosplenial granular cortex; ORB, orbital cortex; PIR, piriform cortex; PVT, paraventricular thalamic nucleus; MD, mediodorsal thalamic nucleus; RT, reticular thalamic nucleus; RE, reuniens thalamic nucleus; VM, ventromedial thalamic nucleus; GPe, globus pallidus, external part; AM, anteromedial thalamic nucleus; LHA, lateral hypothalamic area; VTA, ventral tegmental area; SUM, supramammillary

nucleus; PAG, periaqueductal gray; DR, dorsal raphe nucleus; SC, superior colliculus; MR, median raphe nucleus; IC, inferior colliculus; PPN, pedunculopontine tegmental nucleus; DMH, dorsomedial hypothalamic nucleus; MEA, medial amygdala; COA, cortical amygdala area; NDB, diagonal band; MRN, midbrain reticular nucleus. A, anterior; D, dorsal; L, lateral; M, medial; P, posterior; V, ventral.

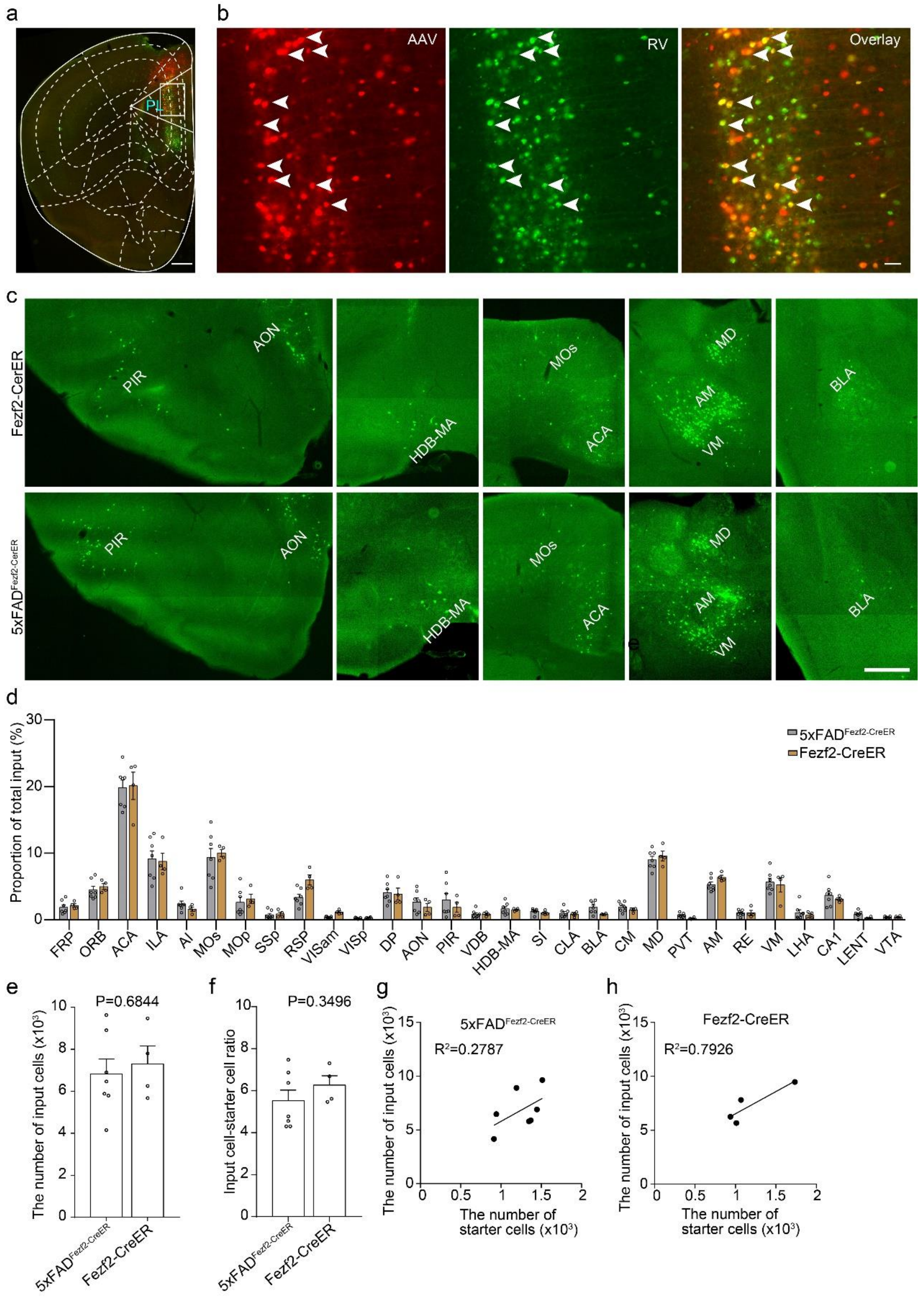

Supplementary figure 12 The whole brain input to extratelencephalic projection neurons in the mPFC of Fezf2-CreER mice and 5×FAD mice at 2 months of age, related to Fig 5. a, b. Representative injection sites showed there was strong helper (red) and rabies (green) virus

expression in the PL. Scale bar: a, 1 mm; b, 100 μm. c. Afferent neurons in different brain areas that established synapses on ET neurons in the mPFC. Scale bar is 1 mm. d. Proportion of whole brain long range inputs to ET neurons in the prefrontal cortex. AD, n=7 animals; WT, n=4 animals. e. Quantification of the total number of the whole brain afferent neurons. AD, n=7 animals; WT, n=4 animals. Two-tailed unpaired t test. f. The input neuron-starter cell ratio in both WT group and the AD group, AD, n=7 animals; WT, n=4 animals. Two-tailed unpaired t test. g, h. The correlation between the number of input neurons and the starter cells in both WT group and the AD group. PL, prelimbic area; PIR, piriform cortex; AON, anterior olfactory nucleus; HDB, horizontal diagonal band; MA, magnocellular nucleus; MOs, secondary motor area; ACA, anterior cingulate area; MD, mediodorsal thalamic nucleus; AM, anteromedial thalamic nucleus; VM, ventromedial thalamic nucleus; BLA, basolateral amygdala; FRP, frontal association cortex; ORB, orbital cortex; ILA, infralimbic area; AI, agranular insular cortex; MOp, primary motor area; SSp, primary somatosensory area; RSP, retrosplenial granular cortex; VISam, anteromedial visual area; VISp, primary visual area; DP, dorsal peduncular cortex; VDB, vertical diagonal band; SI, substantia innominata; CLA, claustrum; CM, central medial thalamic nucleus; PVT, paraventricular thalamic nucleus; RE, reuniens thalamic nucleus; LHA, lateral hypothalamic area; LENT, lateral entorhinal cortex; VTA, ventral tegmental area. All data are listed as the Mean ± SEM.

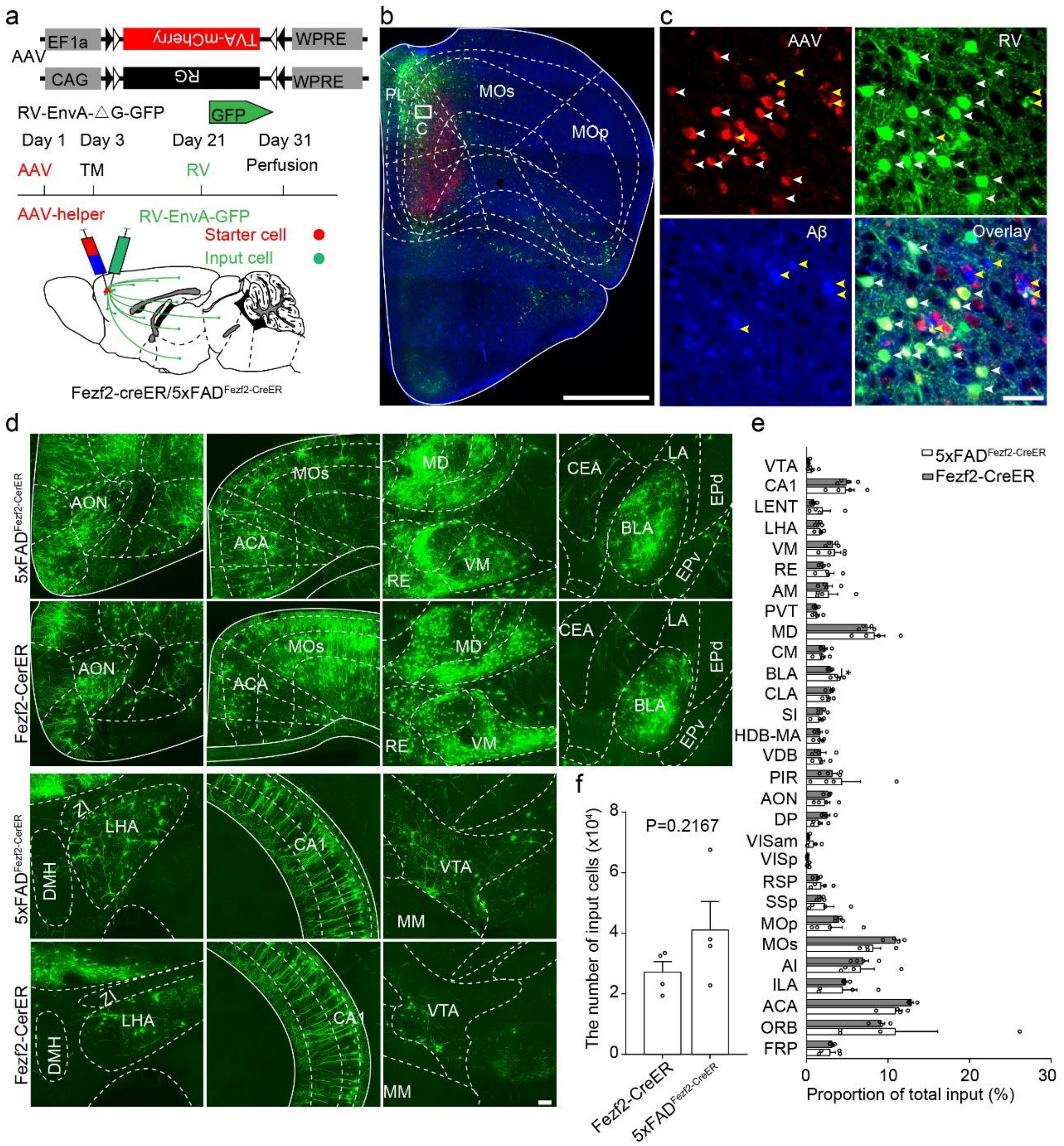

Supplementary figure 13 The whole brain input to extratelencephalic projection neurons in the mPFC of Fezf2-CreER mice and 5×FAD mice at 6 months of age, related to Fig 5. a. AAV helper viruses (AAV-EF1a-DIO-TVA-mCherry and AAV-CAG-DIO-RG) was injected into the PL of $AD_{Fezf2\text{-}CreER}$ or Fezf2-CreER mice at 5 months of age and the Cre recombination was induced with tamoxifen (200 mg/kg) three days after the virus injection. The injection of RV-EnvA-ΔG-GFP was performed 3 weeks after AAV injection. b. Representative injection sites showed there was strong helper (red) and rabies (green) virus expression in the PL. Scale bar: 500 μm. c. Enlarged image boxed in b illustrated some starter neurons and input neurons at the injection site. Scale bar: 50 μm. d. Whole brain datasets acquired by fMOST illustrated neurons in different brain areas that established synapses on ET neurons in the mPFC. Scale bar: 50 μm. e. Proportion of whole brain long range inputs to ET neurons in the prefrontal cortex. f. The total number of input neurons in both $AD_{Fezf2\text{-}CreER}$ group and Fezf2-CreER group. two-tailed unpaired t test, n = 4 animals for each group. For abbreviation, please see Supplementary figure

12. CEA, central amygdala; DMH, dorsomedial hypothalamic nucleus. All data are listed as the Mean ± SEM.

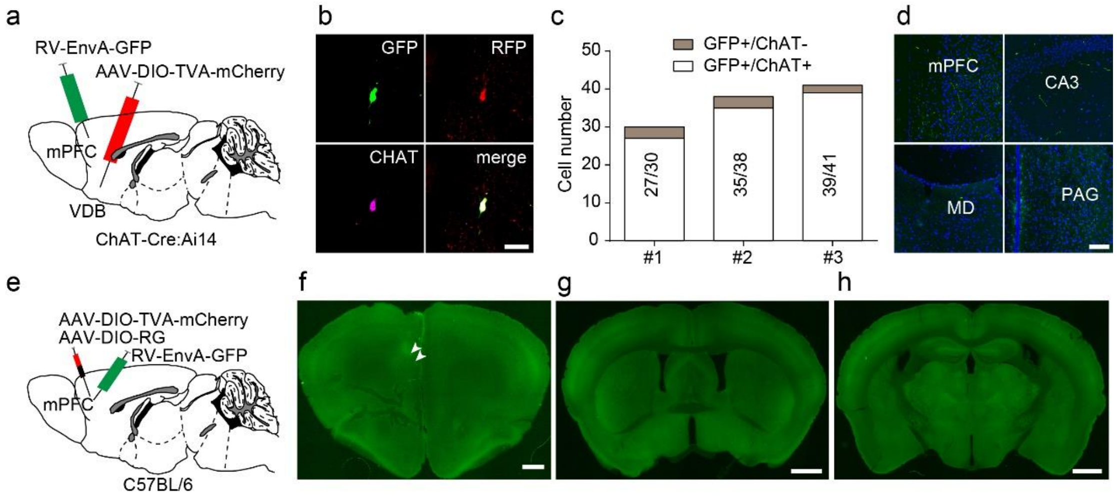

Supplementary figure 14 The specificity of helper virus for RV tracing, related to Fig5. a. AAV-DIO-TVA-mCherry was injected into the VDB of ChAT-Cre mice and the rabies virus coated with EnvA was injected into the mPFC to label the cholinergic neurons in the VDB that project to mPFC. b. Immunochemical staining against choline acetyltransferase to validate the specificity of the helper virus. c. Quantification analysis of the GFP labeled neurons that are cholinergic. d. The collateral projections of the cholinergic neurons in the VDB that project to mPFC. e. The Cre dependent helper virus that express TVA and RG was injected into the mPFC of C57BL/6 mice brain to test the specificity of helper virus. RV was injected into the same site three weeks after the AAV injection. f. The RV labeled neurons at the injection site. g, h. No long-range input neuron was labeled in C57BL/6 mice brain. mPFC, medial prefrontal cortex; MD, mediodorsal thalamic nucleus; PAG, periaqueductal gray. Scale bars in b and d are 50 μm. Scale bars in f, g and h are 1 mm.

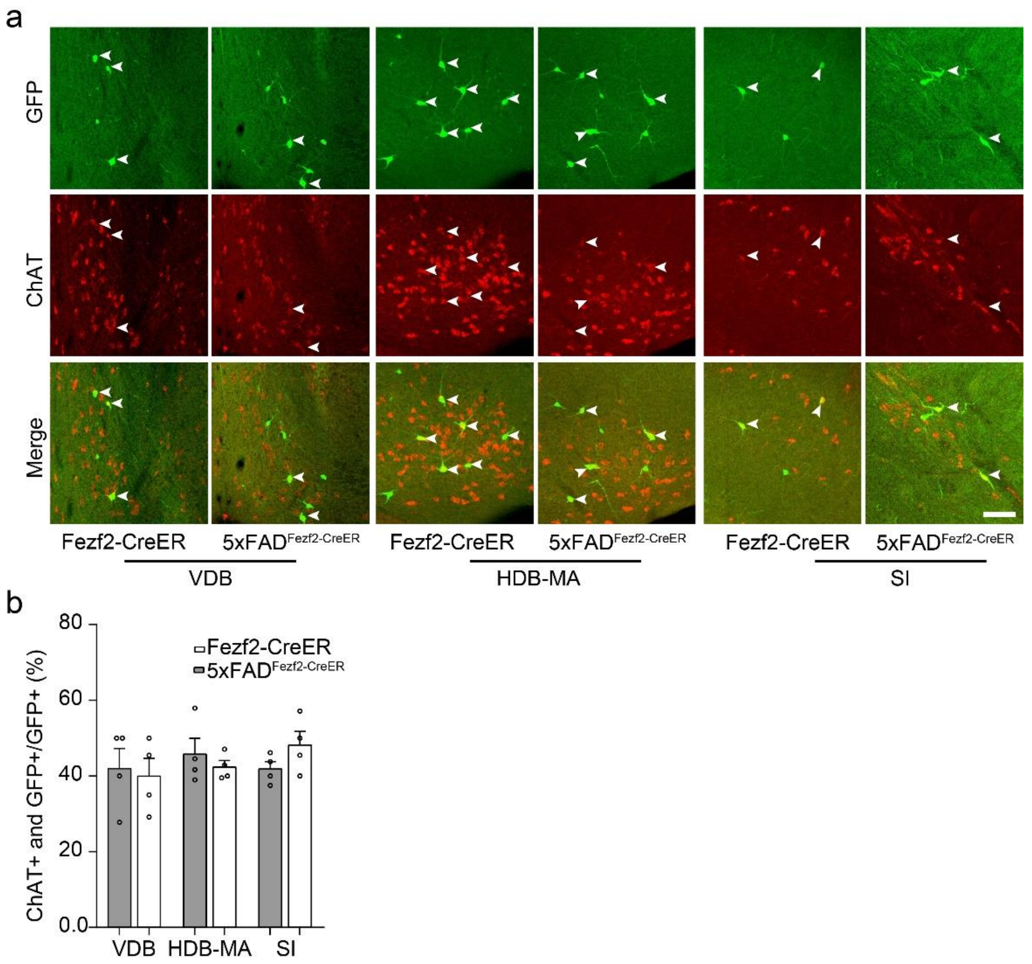

Supplementary figure 15 Cholinergic inputs from basal forebrain to extratelencephalic projection neurons in the mPFC of 5×FAD mice at 2 months of age. a. Immunostaining against ChAT (red) to validate that some of the rabies labeled input neurons (green) from VDB, HDB-MA and SI to extratelencephalic projection neurons in the mPFC are cholinergic neurons. Scale bar: 50 μm. b. Comparison of ChAT positive RV labeled neurons between $AD_{Fezf2\text{-}CreER}$ and Fezf2-CreER mice in VDB, HDB-MA and SI brain area (n = 4 for AD, n = 4 for WT). VDB, vertical diagonal band; HDB, horizontal diagonal band; MA, magnocellular nucleus; SI, substantia innominata. All error bars represent Mean ± SEM.

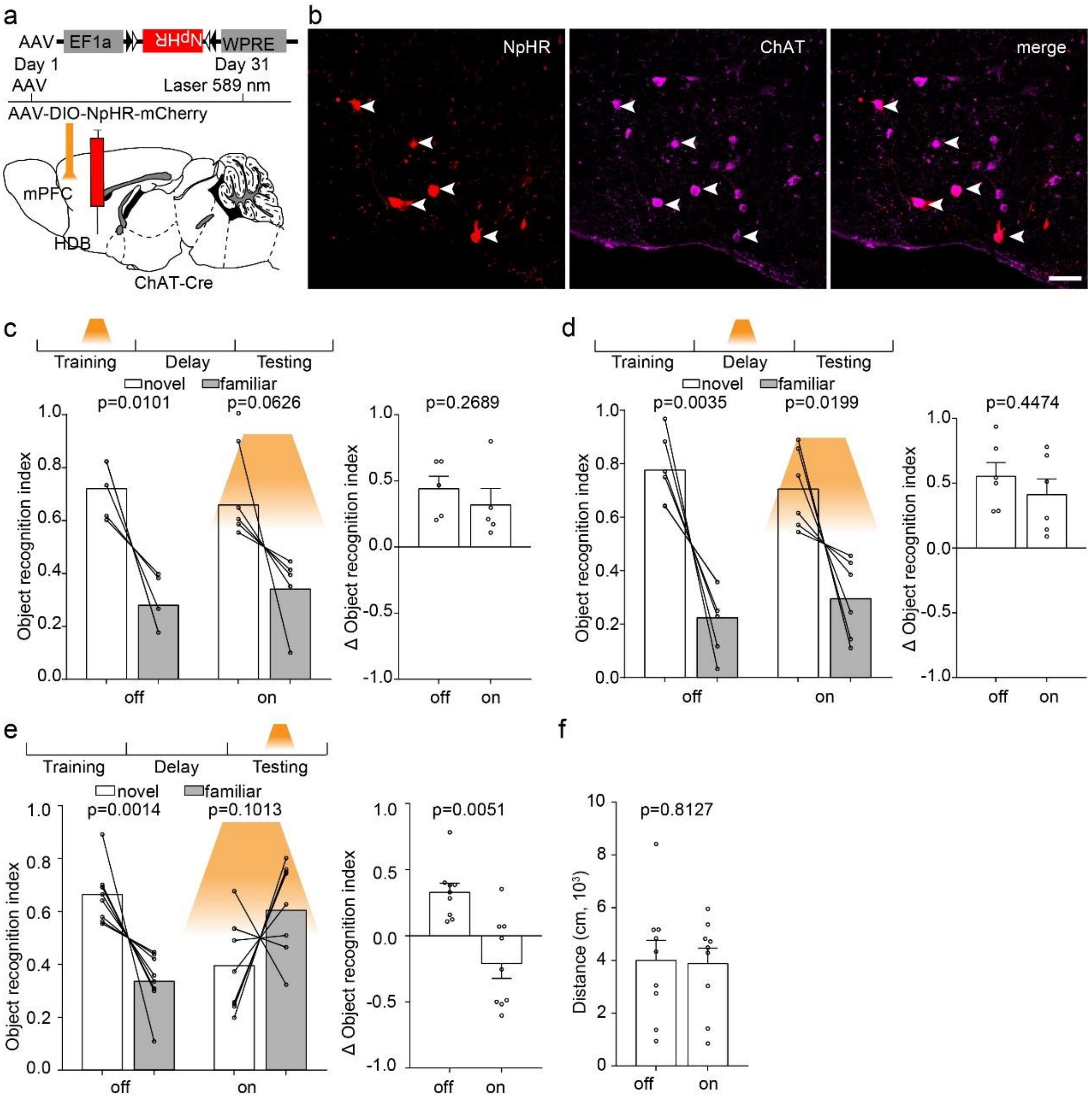

Supplementary figure 16 Inhibition of cholinergic terminals in the mPFC of ChAT-Cre mice in specific phase disrupted object recognition memory, related to Fig 7. a. The experimental strategy of inhibition of cholinergic terminals in the mPFC. The Cre dependent AAV expressing NpHR was injected into the HDB of ChAT-Cre mice. 3-4 weeks after the virus injection, the behavior tests were performed. b. Immunostaining against ChAT to validate the specificity of AAV expression. c. Statistical plots showed that the ChAT-Cre mice tended to explore the novel object under two conditions (with or without light stimulation during training session). The delta object cognition index showed no difference under two conditions. two-tailed paired t test, n = 5 animals. d. Statistical plots showed that the ChAT-Cre mice tended to explore the novel object under two conditions (with or without light stimulation during delay session). The delta object cognition index showed no difference under two conditions. two-tailed paired t test, n = 6 animals. e. Statistical plots showed that during the test session, the ChAT-Cre mice that received light stimulation significantly spent less time exploring the novel object compared to the sessions without light stimulation at 6 months of age. The animals which received light stimulation during test session showed decreased delta object recognition index, two-tailed paired t test, n = 9 animals. f. The statistical plot showed the traveling distance of ChAT-Cre

mice with or without light inhibition of the cholinergic terminals in mPFC. two-tailed paired t test, n = 9 animals. Scale bar in b is 50 μm. All data are listed as the Mean ± SEM.

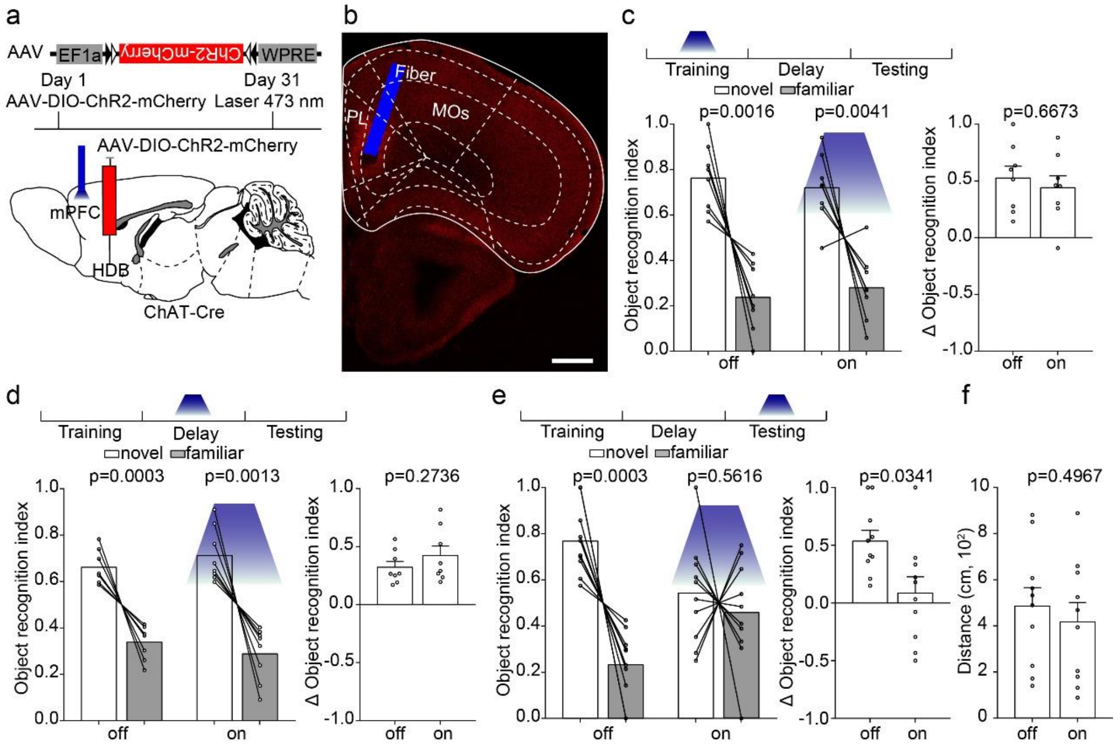

Supplementary figure 17 Activation of cholinergic terminals in the mPFC of ChAT-Cre mice in specific phase disrupted object recognition memory, related to Fig 7. a. The experimental strategy of activation of cholinergic terminals in the mPFC. The Cre dependent AAV expressing ChR2 was injected into the HDB of ChAT-Cre mice. 3-4 weeks after the virus injection, the behavior tests were performed. b. One example image showed the optical fiber position in the mPFC. c. Statistical plots showed that the ChAT-Cre mice tended to explore the novel object under two conditions (with or without light stimulation during training session). The delta object cognition index showed no difference under two conditions. two-tailed paired t test, n = 8 animals. d. Statistical plots showed that the ChAT-Cre mice tended to explore the novel object under two conditions (with or without light stimulation during delay session). The delta object cognition index showed no difference under two conditions. two-tailed paired t test, n = 8 animals. e. Statistical plots showed that during the test session, the ChAT-Cre mice that received light stimulation significantly spent less time exploring the novel object compared to the sessions without light stimulation at 6 months of age. The animals which received light stimulation during test session showed decreased delta object recognition index, two-tailed paired t test, n = 10 animals. f. The statistical plot showed the traveling distance of ChAT-Cre mice with or without light activation of the cholinergic terminals in mPFC. two-tailed paired t test, n=10 animals. Scale bar in b is 500 µm. PL, prelimbic area; MOs, secondary motor area. All data are listed as the Mean ± SEM.

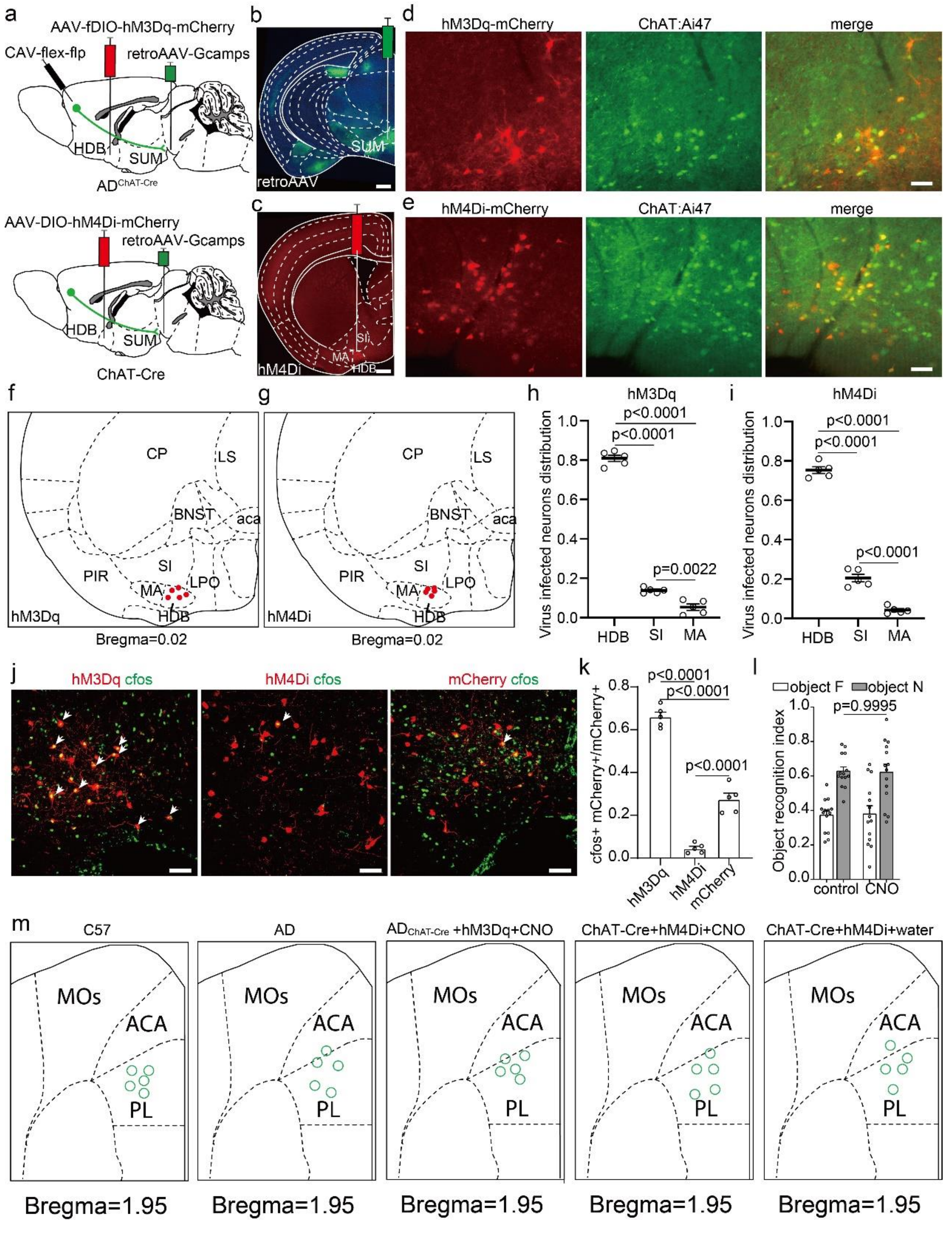

Supplementary figure 18 The specificity and functional validation of AAV expressing hM3Dq and hM4Di, related to Fig 8. a. The experimental strategy to activate or inhibit the mPFC projection cholinergic neurons and to record the activity of SUM projection neurons in the mPFC. For the hM3Dq activation, the flp dependent AAV virus expressing hM3Dq was injected into the HDB of $AD_{ChAT\text{-}Cre}$ mice. Cre dependent CAV expressing flp was injected into the mPFC. The retroAAV expressing GCAMp6s was injected into the SUM to label the SUM projection neurons in mPFC for fiber photometry. For the Hm4Di inhibition, the Cre dependent AAV expressing hM4Di was injected into the HDB of ChAT-Cre mice. The retroAAV expressing

GCAMp6s was injected into the SUM to label the SUM projection neurons in mPFC for fiber photometry. b. The image showed the retroAAV injection site in the SUM. c. The image showed the virus injection site at the HDB. d. The validation of the specificity of the AAV that express hM3Dq and mCherry. The fluorescence of mCherry was highly colocalized with the endogenous expressed GFP. e. The validation of the specificity of the AAV that express hM4Di and mCherry. The fluorescence of mCherry was highly colocalized with the endogenous expressed GFP. f, g. The diagrams showed the injection site of different animals in the HDB. h, i. Quantification of the virus infected neurons around the injection site. n = 5 animals for each group. one-way repeated-measures (RM) ANOVA with Tukey's post hoc test. h. HDB vs SI, $p=6.6\times10^{-13}$; HDB vs MA, $p=2.8\times10^{-13}$. i. HDB vs SI, $p=1.95\times10^{-11}$; HDB vs MA, $p=1.09\times10^{-12}$; SI vs MA, p=0.000022. j. Example images showed the colocalization of c-fos and mCherry in hM3Dq group, hM4Di group and control group after object interaction. k. Statistical plot showed the percentage of colocalization of c-fos and mCherry in hM3Dq group, hM4Di group and control group after object interaction. one-way repeated-measures (RM) ANOVA with Tukey's post hoc test, hM3Dq vs. hM4Di, $p=3.7\times10^{-10}$; hM3Dq vs. mCherry, $p=7.08\times10^{-8}$, hM4Di vs. mCherry, p=0.000018. n = 5 animals for each group. l. Comparison of the effect of chronic administration of CNO alone on object recognition memory. One-way ANOVA followed with Tukey's post hoc test, control group, n=14 animals; CNO group, n=14 animals. m. Schematics showing the placement of optic fibers of fiber photometry experiments in Fig. 8. HDB, horizontal diagonal band; SUM, supramammillary nucleus; MA, magnocellular nucleus; SI, substantia innominata. CP, caudate putamen; LS, lateral septum; aca, anterior commissure, anterior part; BNST, bed nucleus of the stria terminalis; LPO, lateral preoptic area; PIR, piriform cortex; MOs, secondary motor area; ACA, anterior cingulate area; PL, prelimbic area. Scale bars in b and c are 500 μm. Scale bars in d, e and j are 50 μm. All data are listed as the Mean ± SEM.

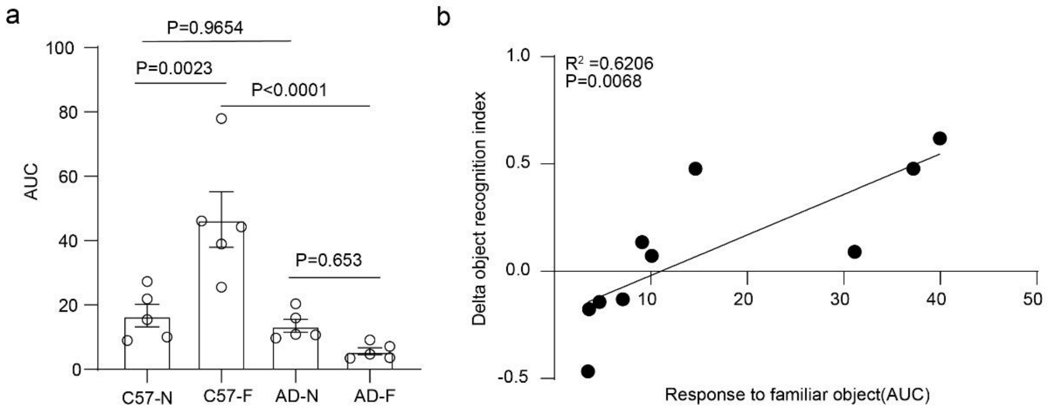

Supplementary fig. 19 The relationship between the object recognition and neuron response to familiar object. a. The ET neuron response in the mPFC of AD animals to familiar but not novel object was decreased compared to that of wild type animals. (one-way ANOVA followed by Tukey correction, C57-F vs AD-F, p=0.0000919. N, novel; F, familiar.). n=5 animals in each group. b. The correlation between the ET neuron response in the mPFC to familiar object and the delta object recognition index. (AD, n=5 animals, wild type, n=5 animals). Linear regression analysis. All data are listed as the Mean ± SEM.

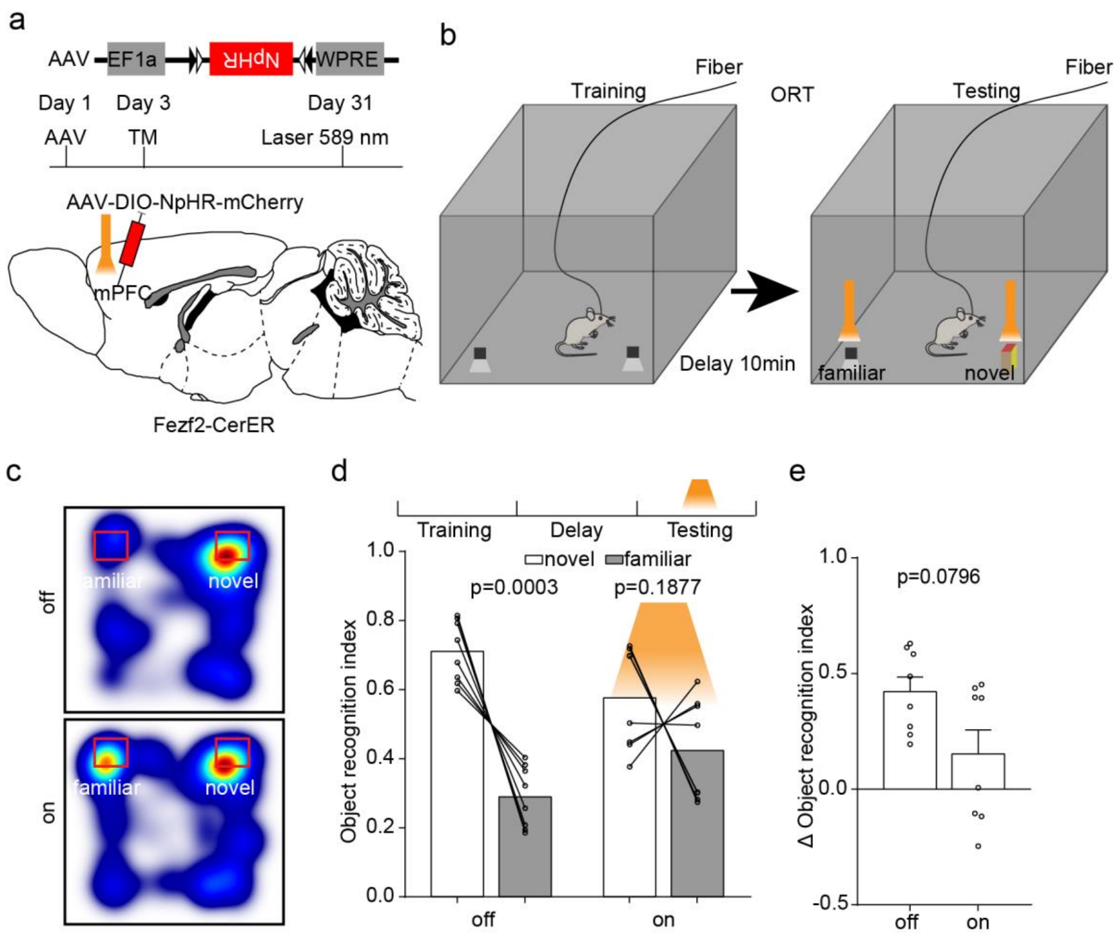

Supplementary figure 20 The effect of region-specific inhibition of extratelencephalic projection neurons in the mPFC during object recognition test. a and b. The experimental procedure to inhibit the extratelencephalic projection neurons in the mPFC and the object recognition test. c. Heat-map plots showed object recognition memory measures from the object recognition test. Red = more time, blue = less time. (upper, without light inhibition; lower, the light inhibition was delivered when animal entered the regions where the objects were located at). d, e. Quantification of the object recognition index and the delta object recognition index of the tests with or without light inhibition. n=8 animals, two-tailed paired t test was used. All data are listed as the Mean ± SEM.

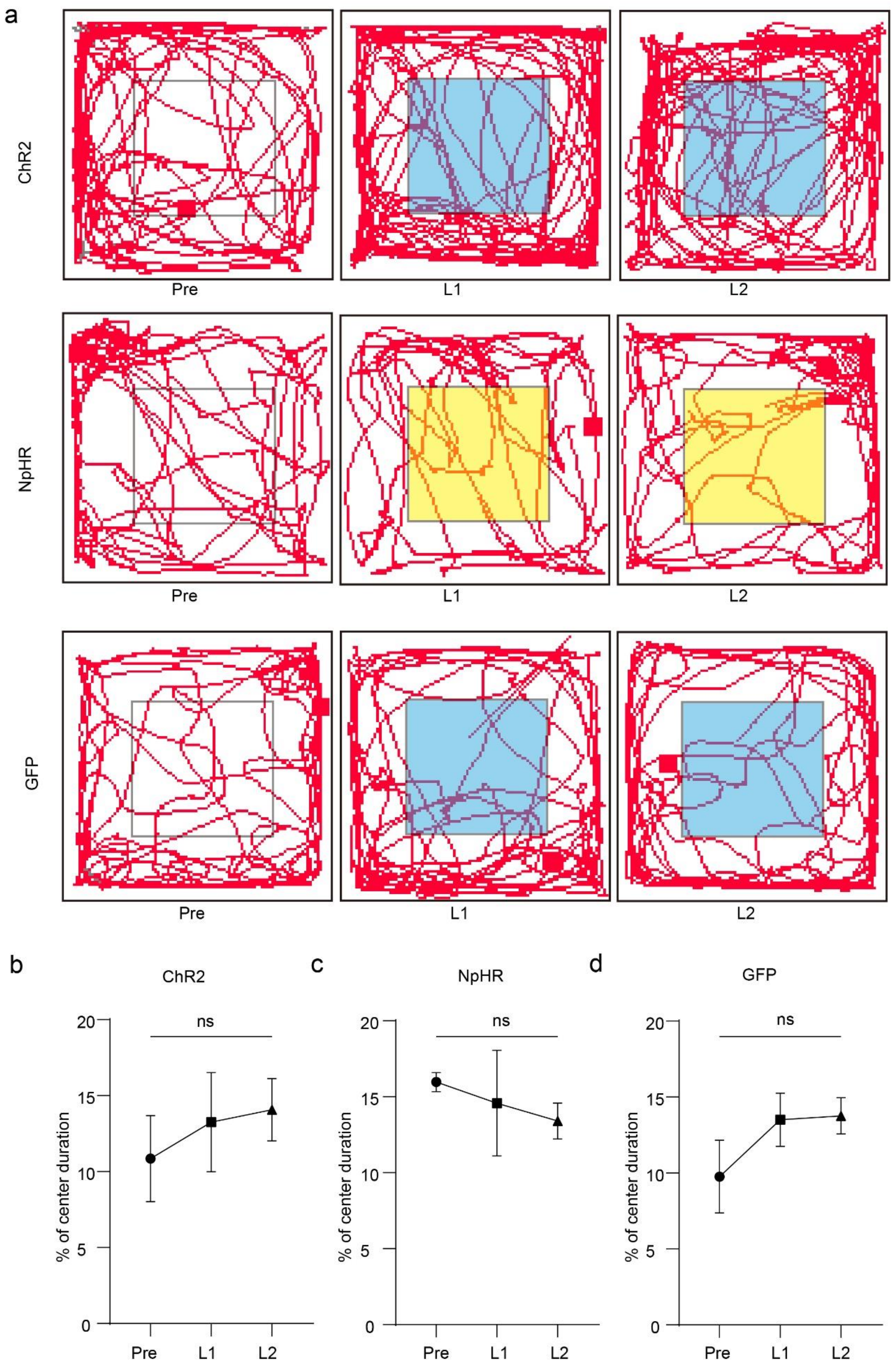

Supplementary figure 21 Optogenetic manipulation of extratelencephalic projection (ET) neurons in the mPFC did not induce robust conditional place preference or aversion. a.

Representative iClass tracks recorded during the iClass test paired with blue light activation or yellow light inhibition in the center of the arena. b. Statistical plot showed that activation of ET neurons in the mPFC during iClass test did not alter the proportion of time that the test mice spent in the center of the area. n = 6 animals. c. Statistical plot showed that inhibition of ET neurons in the mPFC during iClass test did not alter the proportion of time that the test mice spent in the center of the area. n = 5 animals. d. Statistical plot showed that light stimulation of ET neurons in the mPFC that expressed GFP did not alter the proportion of time that the test mice spent in the center of the area. n = 6 animals. one-way repeated-measures (RM) ANOVA with Tukey's post hoc test, all data are listed as the Mean ± SEM.

Supplementary table 1 Probe sequences used for Cre FISH (Highlighted in red).

Cre:

ATGTCCAATTTACTGACCGTACACCAAAATTTGCCTGCATTACCGGTCG
ATGCAACGAGTGATGAGGTTCGCAAG[2]AACCTGATGGACATGTTCAGGGAT
CGCCAGGCGTTTTCTGAGCATACCTGGAAAATGCTTCTGTCCGTTTGCCGG
TCGTGGGCGGCATGGTGCAAGTTGAATAACCGGAAATGGTTTCCCGCAGA
ACCTGAAGATGTTCGCGA[4]TTATCTTCTATATCTTCAGGCGCGCGGTCTGGC
AGTAAAAACTATCCAGCAACATTTGGGCCAGCTAAACATGCTTCATCGTCG
GTCCGGGCTGCCACGACCAAGTGACAGCAATGCTGTTTCACTGGTTATGCG
GCGGATCCGAAAAGAAAACGTTGATGCCGGTGAACGTGCAAAACAGGCTC
TAGCGTTCGA[1]ACGCACTGATTTCGACCAGGTTCGTTCACTCATGGAAAATA
GCGATCGCTGCCAGGATATACGTAATCTGGCATTTCTGGGGATTGC[6]TTATAA
CACCCTGTTACGTATAGCCGAAATTGCCAGGATCAGGGTTAAAGATATCTCA
CGTACTGACGGTGGGAGAATGTTAATCCATATTGGCAGAACGAAAACGCTG
GTTAGCACCGCAGGTGTAGAG[3]AAGGCACTTAGCCTGGGGGTAACTAAACT
GGTCGAGCGATGGATTTCCGTCTCTGGTGTAGCTGATGATCCG[5]AATAACTA
CCTGTTTTGCCGGGTCAGAAAAAATGGTGTTGCCGCGCCATCTGCCACCAG
CCAGCTATCAACTCGCGCCCTGGAAGGGATTTTTGAAGCAACTCATCGATT
GATTTACGGCGCTAAGGATGACTCTGGTCAGAGATACCTGGCCTGGTCTGG
ACACAGTGCCCGTGTCGGAGCCGCGAGATATGGCCCGCGCTGGAGTTT
CAATACCGGAGATCATGCAAGCTGGTGGCTGGACCAATGTAAATATTGTCAT
GAACTATATCCGTAACCTGGATAGTGAAACAGGGGCAATGGTGCGCCTGCT
GGAAGATGGCGATTAG
