# Supplementary material for: Acetylcholine deficiency disrupts extratelencephalic projection neurons in the prefrontal cortex in a mouse model of Alzheimer’s disease
Source: Nat Commun. 2022 Feb 22;13:998. doi: 10.1038/s41467-022-28493-4 (PMC8863829; doi:10.1038/s41467-022-28493-4)
Supplement: Supplementary file 2 — Reporting Summary [file 41467_2022_28493_MOESM2_ESM.pdf]

nature research

Corresponding author(s): Qingming Luo

Last updated by author(s): Dec 7, 2021

Reporting Summary

Nature Research wishes to improve the reproducibility of the work that we publish. This form provides structure for consistency and transparency in reporting. For further information on Nature Research policies, see our Editorial Policies and the Editorial Policy Checklist.

Statistics

For all statistical analyses, confirm that the following items are present in the figure legend, table legend, main text, or Methods section.

n/a	Confirmed	
☐	☒	The exact sample size (*n*) for each experimental group/condition, given as a discrete number and unit of measurement
☐	☒	A statement on whether measurements were taken from distinct samples or whether the same sample was measured repeatedly
☐	☒	The statistical test(s) used AND whether they are one- or two-sided<br>*Only common tests should be described solely by name; describe more complex techniques in the Methods section.*
☒	☐	A description of all covariates tested
☐	☒	A description of any assumptions or corrections, such as tests of normality and adjustment for multiple comparisons
☐	☒	A full description of the statistical parameters including central tendency (e.g. means) or other basic estimates (e.g. regression coefficient) AND variation (e.g. standard deviation) or associated estimates of uncertainty (e.g. confidence intervals)
☐	☒	For null hypothesis testing, the test statistic (e.g. *F*, *t*, *r*) with confidence intervals, effect sizes, degrees of freedom and *P* value noted<br>*Give P values as exact values whenever suitable.*
☒	☐	For Bayesian analysis, information on the choice of priors and Markov chain Monte Carlo settings
☒	☐	For hierarchical and complex designs, identification of the appropriate level for tests and full reporting of outcomes
☒	☐	Estimates of effect sizes (e.g. Cohen's *d*, Pearson's *r*), indicating how they were calculated

Our web collection on statistics for biologists contains articles on many of the points above.

Software and code

Policy information about availability of computer code

Data collection	The softwares and codes used for imaging, image preprocessing, visualization, reconstruction and registration in this study were similar to previous studies(Sun et al. 2019. Nat. Neuroscience, DOI: 10.1038/s41593-019-0429-9; Gong et al. 2016. Nat. Communications, DOI: 10.1038/ncomms12142, Zhong et al. 2021. Nature Methods, doi: 10.1038/s41592-021-01074-x) and described in Supplementary Materials and Methods. The softwares (spike2,CED, Cambridge, UK) and codes used for fiber photometry were described elsewhere(Lin et al. 2020. Neuron; DOI: 10.1016/j.neuron.2020.02.009; Li et al. 2016. Nat. Communications, https://doi.org/10.1038/ncomms10503). Cell counting was performed by ImageJ 2.0 or NeuroGPS(Quan et al. 2013. Scientific Reports, 10.1038/srep01414). Amira software (v 5.2.2, FEI, Me´rignac Cedex, France) was used for data visualization and neuron reconstruction. For behavior tests, EthoVision XT 12.0 (Noldus Apparatus) was used for data collection and analysis. Confocal images were collected via Zen 2011 software of Zeiss.
Data analysis	MatLab (2018a) was used for fiber photometry data analysis. EthoVision XT 12.0 (Noldus Apparatus) was used for data collection and analysis. Cell counting was performed by ImageJ 2.0 or NeuroGPS(Quan et al. 2013. Scientific Reports, 10.1038/srep01414). Statistical analysis was performed using preset algorithms in Graphpad Prism (v.6) . Figures were prepared in Adobe Illustrator CS7.

For manuscripts utilizing custom algorithms or software that are central to the research but not yet described in published literature, software must be made available to editors and reviewers. We strongly encourage code deposition in a community repository (e.g. GitHub). See the Nature Research guidelines for submitting code & software for further information.

Data

Policy information about availability of data

All manuscripts must include a data availability statement. This statement should provide the following information, where applicable:

- Accession codes, unique identifiers, or web links for publicly available datasets
- A list of figures that have associated raw data
- A description of any restrictions on data availability

All the data that support the findings of this study are provided in the article and its Supplementary information files. Source data are provided with this paper. Additional information about this paper are available from the corresponding author upon reasonable request.

Field-specific reporting

Please select the one below that is the best fit for your research. If you are not sure, read the appropriate sections before making your selection.

- [x] Life sciences
- [ ] Behavioural & social sciences
- [ ] Ecological, evolutionary & environmental sciences

For a reference copy of the document with all sections, see nature.com/documents/nr-reporting-summary-flat.pdf

Life sciences study design

All studies must disclose on these points even when the disclosure is negative.

Sample size	No statistical methods were used to predetermine sample size; however, the sample size was similar to previous studies(Yuen, E. Y. et al. 2012, Neuron; Liu, Z. et al. 2014, Neuron; Zhang, Z. et al. 2017, Elife)
Data exclusions	No data were excluded.
Replication	Results described throughout the paper were reproduced. 5-10 rounds of experimentation were performed in independent animals. The n value in the paper indicated the times of replication. No results were included that were not observed in multiple animals. No issues were identified in replicating any of the reported findings.
Randomization	For all experiments in the present study, the animals were randomly assigned to experimental group and control group. For the comparison between AD group and wild type group, the hemizygous 5XFAD mice were randomly assigned to the AD group and the noncarriers of AD mutant genes from same genetic background were randomly assigned to the wild type group.
Blinding	For the behavior experiments including fiber photometry, optogenetics and chemogenetics, the investigator was blinded to the groups. For rabies tracing, the person who analyzed the data was binded to the strain of the animals. For other tracing experiments such supplementary fig 1, the person who performed the experiments was not blinded to the strain of the animals since no comparison was required in those experiments.

Reporting for specific materials, systems and methods

We require information from authors about some types of materials, experimental systems and methods used in many studies. Here, indicate whether each material, system or method listed is relevant to your study. If you are not sure if a list item applies to your research, read the appropriate section before selecting a response.

Materials & experimental systems

n/a	Involved in the study	
[ ]	[x]	Antibodies
[x]	[ ]	Eukaryotic cell lines
[x]	[ ]	Palaeontology and archaeology
[ ]	[x]	Animals and other organisms
[x]	[ ]	Human research participants
[x]	[ ]	Clinical data
[x]	[ ]	Dual use research of concern

Methods

n/a	Involved in the study	
[x]	[ ]	ChIP-seq
[x]	[ ]	Flow cytometry
[x]	[ ]	MRI-based neuroimaging

Antibodies

Antibodies used	For the immunohistochemistry, the mice brain was sectioned at 70-μm thickness by a vibratome (Leica, VS1200S). The coronal brain sections were rinsed with 0.01 M PBS for 3 × 10 min, and blocked with 5% (wt/vol) BSA in 0.01 M PBS (at 37 ℃ for 2h). Next, the brain sections were incubated with the primary antibodies (at 4 ℃ for 12h): anti-c-fos (1:800, rabbit, Cell Signaling Technology, RRID: AB_2247211), anti-Aβ (1:800, mouse, abcam, ab126649), anti-chat (1:200, goat, Millipore, AB144P). Following the incubation with

the primary antibodies, the sections were rinsed with 0.01 M PBS for 3 × 10 min, then incubated with the fluorophore-conjugated secondary antibodies (1:500, at 37 ℃ for 2h): Alexa Fluor-594, donkey anti goat; Alexa Fluor-405, goat anti mouse; Alexa Fluor-594 or 647, donkey anti rabbit. Following this procedure, the brain sections were rinsed with 0.01 M PBS for 3 × 10 min, and finally the brain sections were attached to a glass slide and imaged with a commercial confocal microscope (Carl Zeiss, LSM710) or a slide scanner (Olympus VS120). The information of the secondary antibodies can be found in the following link:
Goat mouse Invitrogen Alexa Fluor 405 A-31553 "https://www.thermofisher.com/antibody/product/Goat-anti-Mouse-IgG-H-L-Cross-Adsorbed-Secondary-Antibody-Polyclonal/A-31553"
Donkey anti-Goat IgG (H+L) Highly Cross-Adsorbed Secondary Antibody, Alexa Fluor Plus 594: https://www.thermofisher.com/antibody/product/Donkey-anti-Goat-IgG-H-L-Highly-Cross-Adsorbed-Secondary-Antibody-Polyclonal/A32758
Donkey anti-Rabbit IgG (H+L) Highly Cross-Adsorbed Secondary Antibody, Alexa Fluor 647: https://www.thermofisher.com/antibody/product/Donkey-anti-Rabbit-IgG-H-L-Highly-Cross-Adsorbed-Secondary-Antibody-Polyclonal/A-31573
Donkey anti-Rabbit IgG (H+L) Highly Cross-Adsorbed Secondary Antibody, Alexa Fluor 594: https://www.thermofisher.com/antibody/product/Donkey-anti-Rabbit-IgG-H-L-Highly-Cross-Adsorbed-Secondary-Antibody-Polyclonal/A-21207

Validation

These are all well characterized commercial antibodies.The specificity of the primary and secondary antibodies was validated by the manufacturers.
cfos: https://www.cellsignal.com/products/primary-antibodies/c-fos-9f6-rabbit-mab/2250?site-search-type=Products&N=4294956287&Ntt=cfos&fromPage=plp
Aβ: https://www.abcam.com/beta-amyloid-antibody-moab-2-ab126649.html
chat: https://www.emdmillipore.com/US/en/product/Anti-Choline-Acetyltransferase-Antibody,MM_NF-AB144P
Goat mouse Invitrogen Alexa Fluor 405 A-31553 "https://www.thermofisher.com/antibody/product/Goat-anti-Mouse-IgG-H-L-Cross-Adsorbed-Secondary-Antibody-Polyclonal/A-31553"
Donkey anti-Goat IgG (H+L) Highly Cross-Adsorbed Secondary Antibody, Alexa Fluor Plus 594: https://www.thermofisher.com/antibody/product/Donkey-anti-Goat-IgG-H-L-Highly-Cross-Adsorbed-Secondary-Antibody-Polyclonal/A32758
Donkey anti-Rabbit IgG (H+L) Highly Cross-Adsorbed Secondary Antibody, Alexa Fluor 647: https://www.thermofisher.com/antibody/product/Donkey-anti-Rabbit-IgG-H-L-Highly-Cross-Adsorbed-Secondary-Antibody-Polyclonal/A-31573
Donkey anti-Rabbit IgG (H+L) Highly Cross-Adsorbed Secondary Antibody, Alexa Fluor 594: https://www.thermofisher.com/antibody/product/Donkey-anti-Rabbit-IgG-H-L-Highly-Cross-Adsorbed-Secondary-Antibody-Polyclonal/A-21207

Animals and other organisms

Policy information about studies involving animals; ARRIVE guidelines recommended for reporting animal research

Laboratory animals

The 5xFAD mice line express human APP and PSEN1 transgenes with a total of five FAD mutations: KM670/671NL (Swedish), I716V (Florida), and V717I (London) mutations in APP, and M146L (A>C) and L286V mutations in PSEN1 has been described(Oakley, H. et al. 2006, Journal of Neuroscience). C57BL/6J, ChAT-ires-Cre mice (stock No: 018957), and 5xFAD mice (stock No: 034848) were purchased from Jackson Laboratory (Bar Harbor, ME, USA). Fezf2-CreER mice was a gift from Josh Huang lab. Ai47 reporter line was a gift from Hongkui Zeng of the Allen Institute for Brain Science. Heterozygous male 5×FAD mice was crossed with Fezf2-CreER females and ChAT-Cre mice, respectively, and the progeny were genotyped by PCR. The female 5×FAD mice, 5×FADFezf2-CreER mice and 5×FADChAT-Cre at 2, 5 and 6 months of age and the same age of WT (C57, Fezf2-CreER or ChAT-Cre) were used for experimental studies. All animals were housed in a room under the conditions of 21±1℃ (humidity: 40%-70%) and 12-h light/dark cycle with lights on at 8:00 am with food and water ad libitum. Female animals were employed for all the animal experiments.

Wild animals

The study did not involve wild animals.

Field-collected samples

The study did not involve data collected from the field.

Ethics oversight

All animal experiments were approved by the Animal Care and Use Committee of Huazhong University of Science and Technology.

Note that full information on the approval of the study protocol must also be provided in the manuscript.
